# Self-reported childhood family adversity is linked to an attenuated gain of trust during adolescence

**DOI:** 10.1038/s41467-023-41531-z

**Published:** 2023-10-30

**Authors:** Andrea M. F. Reiter, Andreas Hula, Lucy Vanes, Tobias U. Hauser, Danae Kokorikou, Ian M. Goodyer, NSPN Consortium, Peter Fonagy, Michael Moutoussis, Raymond J. Dolan

**Affiliations:** 1 Max Planck UCL Centre for Computational Psychiatry and Ageing Research, University College London, London, UK; 2 Wellcome Centre for Human Neuroimaging, University College London, London, UK; 3 Department of Child and Adolescence Psychiatry, Psychosomatics and Psychotherapy, Centre of Mental Health, University Hospital Würzburg, Würzburg, Germany; 4 Department of Psychology, Julius-Maximilians-Universität Würzburg, Würzburg, Germany; 5 CRC Cognitive Control, Faculty of Psychology, Technische Universität Dresden, Dresden, Germany; 6 Austrian Institute of Technology, Vienna, Austria; 7 Department of Neuroimaging, Institute of Psychiatry, Psychology & Neuroscience, King’s College London, London, UK; 8 Department of Psychiatry and Psychotherapy, Medical School and University Hospital, Eberhard Karls University of Tübingen, Tübingen, Germany; 9 German Center for Mental Health (DZPG), Tübingen, Germany; 10 Department of Clinical, Educational and Health Psychology, University College London, London, UK; 11 Department of Psychiatry, University of Cambridge, Cambridge, UK; 12 State Key Laboratory of Cognitive Neuroscience and Learning, IDG/McGovern Institute for Brain Research, Beijing Normal University, Beijing, China

**Keywords:** Human behaviour, Decision, Social behaviour

## Abstract

A longstanding proposal in developmental research is that childhood family experiences provide a template that shapes a capacity for trust-based social relationships. We leveraged longitudinal data from a cohort of healthy adolescents (*n* = 570, aged 14–25), which included decision-making and psychometric data, to characterise normative developmental trajectories of trust behaviour and inter-individual differences therein. Extending on previous cross-sectional findings from the same cohort, we show that a task-based measure of trust increases longitudinally from adolescence into young adulthood. Computational modelling suggests this is due to a decrease in social risk aversion. Self-reported family adversity attenuates this developmental gain in trust behaviour, and within our computational model, this relates to a higher ‘irritability’ parameter in those reporting greater adversity. Unconditional trust at measurement time point T1 predicts the longitudinal trajectory of self-reported peer relation quality, particularly so for those with higher family adversity, consistent with trust acting as a resilience factor.

## Abstract (web summary)

The authors examined how family experiences are linked to the development of trust in adolescence and young adulthood. They show that trust increases over time but self-reported family adversity can hinder this, and trust may act as a resilience factor in maintaining positive peer relations.

## Introduction

Family experience provides a foundation for effective later life social functioning. This is particularly important in adolescence when other-oriented behaviours mature, leading to the establishment and maintenance of stable relationships^1–3^. The emergence of trust is considered an important contributory factor to such other-oriented behaviours, where trust is defined as a willingness to be vulnerable to the actions of another party^4,5^. This willingness encapsulates a positive expectation that the reciprocal actions of another will be beneficial, irrespective of one’s own ability to exert control over the other. As such, trust is commonly conceived as incorporating a risk-benefit trade-off, where trusting another entails uncertainty both to potential negative consequences but also positive consequences.

Economic games are a commonly deployed means for quantifying trust, enabling measurement of inter-individual differences in the risk-benefit trade-off for trust, including an ability to establish and maintain cooperative behaviour^6,7^. This quantitative approach has helped identify neural correlates of trust-based decision-making, implicating activity in anterior insula^8,9^ (for meta-analysis). Computational modelling of economic trust games^10,11^ has also suggested that core cognitive mechanisms underlying trust behaviour include social preference factors (a willingness to tolerate social risk, social inequity aversion), a capacity to plan ahead as well as an awareness of the states of mind of self and others (see Table 1 for an overview and conceptual description). Importantly, a parameter in the model captures an agent’s irritable state, formalising retaliatory impulses induced by perceived unfairness (‘Irritability’)^10,11^.

**Table 1:** Computational Model of investment behaviour in the multi-round trust task: Parameters, ranges and conceptual description of the parameters

Parameter symbol	Parameter name	Possible parameter values	Meaning
${{{{{\boldsymbol{\alpha }}}}}}$	Inequality aversion	$\left\{{0,\, 0.4,\, 1}\right\}$	Degree of sensitivity to an unfair outcome against the other player.
${{{{{\boldsymbol{\omega}}}}}},\, {{{{{\boldsymbol{b}}}}}}^{{{{{\boldsymbol{T}}}}}} \, ({{{{\boldsymbol{\omega}}}}})$	Risk Aversion	$\left\{{0.4,\, 0.6,\, 0.8,\, 1,\, 1.2,\, 1.4,\, 1.6,\, 1.8 }\right\}$	Multiplier for value of money kept over money returned by the partner.
${{{{{\boldsymbol{k}}}}}}$	Theory of Mind sophistication	$\left\{{0,\, 1,\, 2,\, 3,\, 4}\right\}$	Number of recursive reasoning steps in representing beliefs of the other player.
${{{{{\boldsymbol{P}}}}}}$	Planning	$\left\{{1,\, 2,\, 3,\, 4}\right\}$	Number of steps ahead planned into the interaction.
${{{{{\boldsymbol{\zeta }}}}}}$	Irritability	$\left\{0,\, 0.25,\, 0.5,\, 0.75,\, 1\right\}$	Measure of shift towards punishment behaviour, when experiencing below expectation partner actions.
${{{{{\bf{q}}}}}}{{{{{\boldsymbol{(}}}}}}{{{{{\boldsymbol{\zeta }}}}}}{{{{{\boldsymbol{)}}}}}}$	Irritation Awareness	$\left\{0,\, 1,\, 2,\, 3,\, 4\right\}$	Awareness of partner irritability. 0 = unaware, 4 = partner for sure irritable.
${{{{{\boldsymbol{\beta }}}}}}$	Inverse Temperature	$\left\{\frac{1}{4},\, \frac{1}{3},\, \frac{1}{2},\, \frac{1}{1}\right\}$	Measure of stochasticity in choices given their expected utilities.

Economic trust games have primarily been deployed in cross-sectional studies, with inconsistent findings especially in relation to a mid- and late adolescent period. Some studies, including a previously reported analysis of baseline data from the current study^12^, show age-related increases in trust^12–14^, while others find no age-related differences^15–17^ or even decreases during the course of adolescence^18^. Such inconsistencies may, in part, reflect substantial inter-individual differences in development, a factor that cannot be examined in studies that deploy cross-sectional designs. Indeed, previous research in adolescent neurocognitive development has concentrated largely on ‘average’ development^19^, potentially concealing meaningful inter-individual variability in social development. It is widely considered that longitudinal designs are necessary to fully capture change at an individual level^20–22^.

An important source of inter-individual variability in adolescent social development is the impact of social experiences occurring during earlier developmental periods,  which may cascade across successive developmental phases^23^. Distinct developmental phases carry their own unique social challenges – from engagement with a caregiver during infancy to integration with a peer group in adolescence. Indeed, a long-standing hypothesis in developmental psychopathology proposes that earlier childhood family experiences, including parent-child interactions, are critical constituents in the emergence of an ability to establish and maintain healthy later life social relationships^24–27^.

In this study we detail typical longitudinal developmental trajectories of trust, including underlying cognitive mechanisms, with specific attention to characterising inter-individual differences in developmental trajectories of trust. Based on the literature, we focused on a hypothesis that past family experiences shape how trust develops intra-individually during adolescence. Based on self-reported family and parenting experiences we derived a family experience factor^28^, which we hypothesized would predict individual adolescent trust development. To ascertain the relevance of trust for real-life social relationships, we examined how inter-individual differences in trust related to the development of peer relations.

We show, both cross-sectionally and longitudinally, that there is an increase in trust from adolescence into young adulthood. Computational modelling on our data provides insight into this normative developmental process, showing the emergence of trust is best explained by reference to an adaptive decrease in social risk aversion over the course of adolescence, which confers adaptive benefits in task performance. Notably, self-reported family adversity attenuates the observed longitudinal gain in trust, while unconditional trust in our economic game predicts the quality of future social relationships, particularly so in those with a history of pronounced self-reported adversity.

## Results

**Fig. 1:**
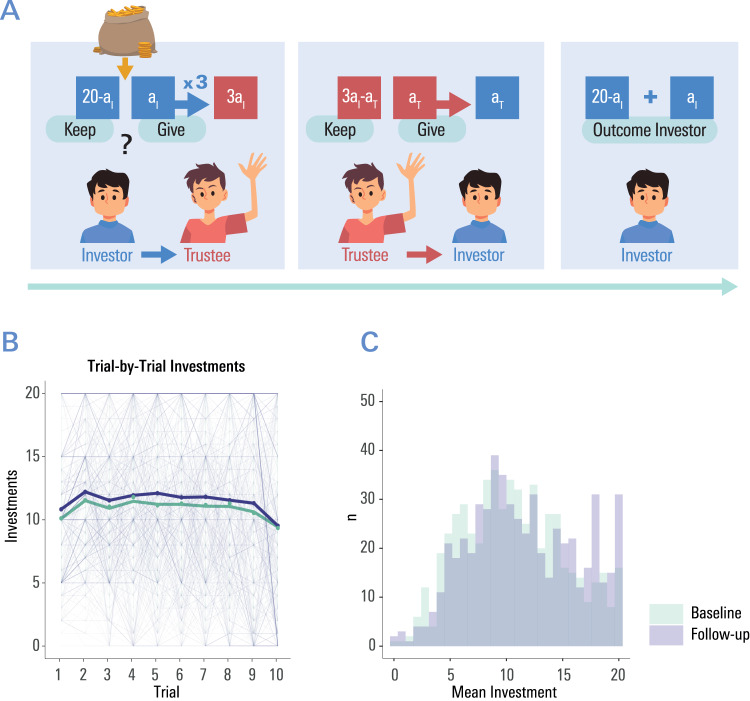
Assessing Trusting Behaviour in a Multiround Trust Game. (A) Illustration of one round in the trust game. At the beginning of each round, the participant (adopting the role of the ‘investor’) received 20 play-coins. The participant decided how much of this credit to invest with another player (‘trustee’), and how much they wanted to keep for themselves. The participant was informed that their investment a_i_, (but not the money they kept) would be tripled by the experimenter. In a next step, the trustee (unbeknownst to the participant, a computer algorithm which was informed by trustee decisions in previous studies), decided on the amount of coins to repay to the participant (a_T_). Participants were shown the trustee’s repayment a_T_ at the end of each round. Coins gained were not transferred to the next round, which would start with a credit of 20 coins again. Participants played a total of 10 rounds. **B** Amount of coins invested in round 1 to 10; thicker line denotes mean investment as a function of time (T1 vs. T2). **C** Histogram of mean investments across all rounds at baseline (T1) and after the ~1.5 years follow-up period (T2).

We focus on longitudinal analyses (~1.5-year follow-up), involving multi-modal measurements, across 570 participants (14–25 years of age, 284 female, 286 male (sex assessment based on participants’ self-report, see Methods)). A cross-sectional analysis based on the baseline dataset has been published previously^12^. To assess trusting behaviour, participants engaged in 10 rounds of an interactive multi-round trust game^7,12^ (Fig. 1A), in which they played the role of the so-called investor. In each of ten rounds, the investor received an initial credit of 20 coins, and then decided how much of this credit to transfer to another person (the trustee; unbeknownst to our participants, mimicked by a computer algorithm). The participant was informed that their investment would be tripled by the experimenter before the trustee then decides how many coins to pay back to the investor. Participants were shown the trustee’s repayment at the end of each round.

To gain insight into the development of different aspects of trusting behaviour, as measured in our iterative economic task, we ran a series of complementary analyses. First, we analysed the amount invested in the first round as an index of a priori, initial (i.e., unconditional) trust, before any interaction with a partner had occurred. Secondly, we took account of all ten rounds that a participant played and analysed round-by-round investments as an index of mean trust. Note that while the latter analysis takes account of all repeated decisions made by one individual, these are biased by the interaction experienced. Lastly, we derived a measure of reciprocity, based on prior literature^6^, defined as relative change in investment from one round to another in response to a change in repayment by the partner on a previous round. This definition is de facto an operationalisation of tit-for-tat like behaviour^6^ (see SI-Methods for details).

### Behavioural analyses: longitudinal development of trust behaviour

We used linear mixed effects models to ascertain whether trust-related behaviours (first round investment, round-by-round investment, reciprocity) show age-related and within-person developmental change over a ~1.5 year follow-up period by analysing them against cross-sectional (mean) age and longitudinal developmental effects (‘longitudinal age’, see Methods for details).

We found both cross-sectional age effects (F(1,567.00) = 19.10, *p* < 0.001, estimate = 0.26, se = 0.06), as well as longitudinal effects (F(1,568.00) = 19.45, *p* < 0.001, estimate = 0.67, se = 0.15), on investment behaviour in the first round of the game (i.e., a priori trust). This indicates that a-priori trust increases both between persons with increasing age, as well as within persons with development. There was no evidence for an interaction of longitudinal development and cross-sectional age effects (estimate = −0.03, sd = 0.05, F(1,568.00) = 0.320, *p* = 0.572, see S-Table 1 for full output of the model).

**Fig. 2:**
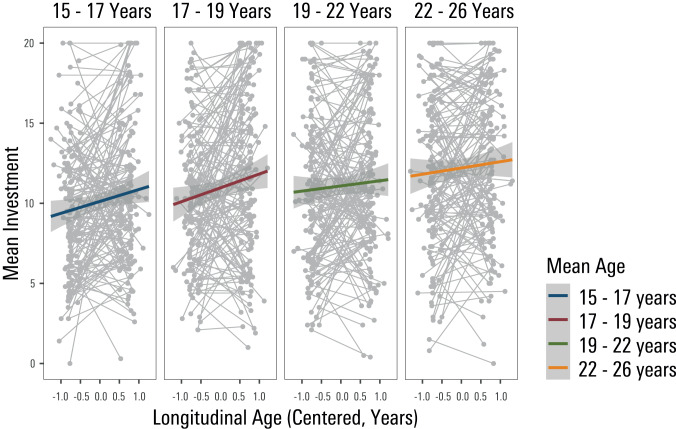
Longitudinal increase of trusting behaviour as a function of participants’ age. Illustration of participants’ mean round-by-round investments in their counterpart (trustee), used as an index of trust, increased with age as well as with longitudinal development. Longitudinal increase in trust (i.e., mean investments) is steepest for the youngest of the sample. For visualisation purposes alone, age is split up into four categories (based on quantiles) in the figure, whereas age entered the statistical model as a continuous regressor. Longitudinal age is mean-centred. The error band shows the standard error of the estimated mean response at each level of age.

**Fig. 3:**
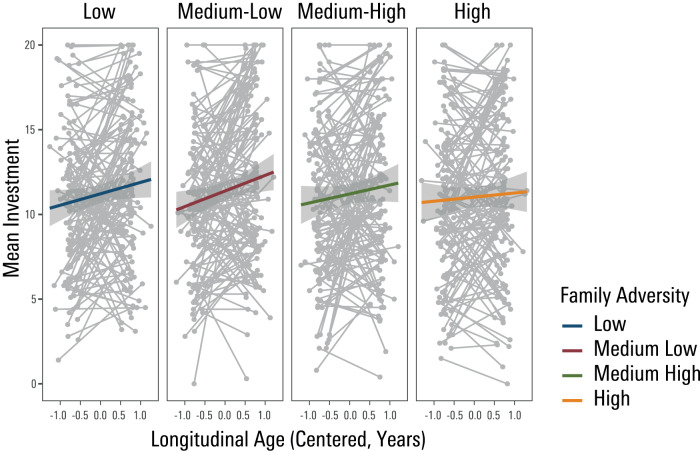
Significant interaction of family experiences and longitudinal development on investment trust behaviour. The development of trust, indexed by round-by-round investment behaviour, is flattened in those who report a higher level of family adversity. Note that whilst family experiences entered the model as a continuous regressor, we categorize it (based on quartiles) for visualization purposes. Longitudinal age is mean-centred. The error band shows the standard error of the estimated mean response at each level of family adversity.

Both cross-sectional age (F(1,567.00) = 24.32 *p* < 0.001, estimate = 0.58, se = 0.06) and longitudinal development (F(1,10819.00) = 109.81, *p* < 0.001, estimate = 0.27, se = 0.05) were statistically significant predictors of round-by-round investment behaviour (see S-Table 2 for full output of the model). This indicates that mean trust increased significantly with age in a between-subject manner, but critically this was also evident as a within-person effect (see Fig. 2). The interaction of longitudinal development and cross-sectional age was statistically significant (F(1,10819) = 15.14, *p* < 0.001, estimate = −0.07,  se= 0.02), implying the extent to which investment behaviour changed longitudinally from baseline to follow-up was dependent on the age of the participants. In essence, a longitudinal increase of trust was steepest for the youngest of the sample whereas the developmental increase in mean trust flattened in older adolescents/early adults (see Fig. 2). A supplementary analysis on a subgroup of participants who underwent the task three times (retest sample, *n* = 55 who played the task ~6 months after the first task assessment) was conducted (see S-Fig. 3 and^29,30^ for details on this subsample analysis). Testing for an effect of the factor time point (baseline, short follow-up, long follow-up) on round-by-round investments in the subsample that had completed all three measurements, revealed a statistically significant effect of time point (F(2,1418.00) = 4.77, *p* = 0.009). Critically, post-hoc analyses showed that investment behaviour increased significantly over the 18-month period (*t* = 2.74, *p* = 0.006), and from the 6-month to the 18-month period (*t* = 2.61, *p* = 0.009) but importantly did not do so significantly over the 6-month period from baseline (*t* = 0.13, *p* = 0.90). This pattern was consistent with longitudinal effects being more likely to be explained by developmental, as opposed to retest, effects.

We found no credible evidence for cross-sectional age or longitudinal developmental effects on reciprocity (all Fs <0.086, all ps>0.05). Here we formalise reciprocity in terms of the relative change in investment from one round to another in response to a change in repayment by a partner, based on the literature^6^. See SI for results on complementary reciprocity measures analysed.

### Computational modelling

Next, we turned to computational modelling (see Methods for details) to probe cognitive mechanisms underlying trust development. Here we availed of a model, validated for this multi-round trust task^11,12^, that describes how an agent observes a partner’s behaviour and then tries to infer the partner’s key characteristics. Thus, an agent considers what their partner believes about them, and, if they have sufficient theory of mind, what the partner believes about their own beliefs. They also take account of what the partner is likely to make of their own behaviour in the next few rounds, allowing them to signal to the partner. For example, by avoiding investment reductions they might want to avoid the possibility of rendering the partner irritable (see methods and^11^ for details and formal description of the model). Thus, in this model, investment decisions as a function of trustee repayment are captured by seven parameters; namely Social Risk Aversion, Theory of Mind, Planning, Guilt, Irritability, Irritation Awareness and Stochasticity (see methods, Table 1 and^11^ for details). We previously validated this computational modelling approach in the current sample^12^, where the focus was on inter-individual differences on model parameters in the baseline (T1) dataset alone.

The sole parameter showing age- or developmental effects (Bonferroni-correction for multiple comparisons applied) was social risk aversion (all other Fs < 2.20, ps > 0.14). As defined by our computational model, social risk aversion denotes a preference for play coins that were not invested over and above play coins that were returned by the trustee. Thus, it expresses a subjective devaluation of play coins invested and re-payed by the partner, due to an inherent risk of coins not being returned by the trustee. Longitudinal modelling revealed a statistically significant effect of both cross-sectional age (F(1, 566.92) = 20.50, *p* < 0.001, estimate = −0.21, se = 0.004) as well as longitudinal development (F(1, 567.10) = 20.49, *p* < 0.001, estimate = −0.055, se = 0.01) on this social risk aversion parameter. This indicates that social risk aversion decreased from adolescence to adulthood, both between-subject (with age) as well as within-subject (longitudinally). The interaction of cross-sectional age and longitudinal age was not statistically significant (F(1, 567.35) = 2.36, *p* = 0.125, estimate = 0.006, se = 0.004).  This indicates no evidence for longitudinal change varying significantly as a function of participants’ age. Notably, social risk aversion lead to reduced total wins at the end of each testing session, both at T1 (t(785) = −25.65, *p* < 0.001, *r* = −0.68), and at follow-up (t(567) = −26.85, *p* < 0.001, *r* = −0.75, see S-Fig. 2). Next, we analysed the effect of the factor time point (baseline, short follow-up, long follow-up) on social risk aversion in the retest sample. We observed a statistically significant effect of measurement time point (F(2,106) = 4.11, *p* = 0.02, S-Fig. 3b). Post-hoc analyses revealed that social risk aversion decreased significantly over the 18-month period (*t* = 2.86, *p* = 0.005), but not significantly during the shorter periods (no statistically significant effect from baseline to 6-month follow-up (*t* = 1.23, *p* = 0.22), and from the 6-month to the 18-month follow up (t(106) = 1.63, *p* = 0.11)).

**Fig. 4:**
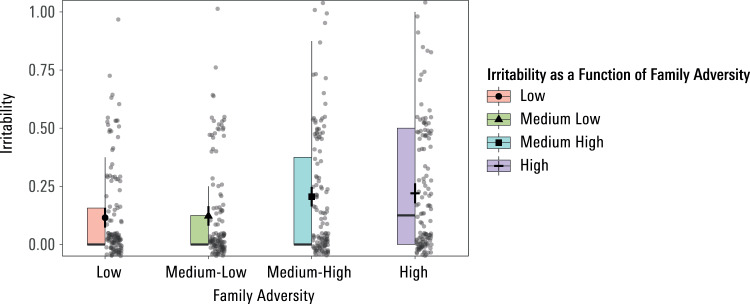
Positive association of family adversity and irritability across both measurement time points. We plot posterior estimates and model-based error bars (95% confidence intervals) derived from the linear mixed model analysis (*n* = 511 participants) using the afex_plot function in R^69^. The level of self-reported family adversity is categorized for visualisation purposes only. Whilst many participants show a computational Irritability parameter = 0, i.e., do not show this form of un-mentalized retaliation at all, it can be seen that those with higher family adversity are over-represented with respect to higher Irritability values. The box represents the middle 50% of the data, the line drawn through the box represents the median, the symbols represent the mean. The lower and upper edges of the box represent the first and third quartiles, respectively.

There is a conflicting literature on whether adolescents are generally more, equally, or less risk-taking than other age groups in non-social contexts (see^31^ for meta-analysis). Thus, we analysed whether age differences on our social risk parameter were explained by more conventional measures of risk preference in a traditional, non-social gambling paradigm^32,33^ which we had also assessed at T1 as part of the behavioural task battery (see S-Fig. 4 and^33^ for details of the task). We found no statistically significant evidence for associations of the proportion of trials participants gambled, nor with more formal risk preference parameters (variance or skewness preference^32^), with age (correcting for multiple comparisons, all ps > 0.060, see S-Fig. 4). Further, the effect of age on social risk aversion at T1 remained statistically significant when including non-social gambling as a covariate into the regression model (*t* = −5.180, *p* < 0.001, estimate = −0.024, se = 0.005,). Thus, an association of age and risk aversion only emerged in the case of the social trust paradigm, but not on more conventional risk taking measures. This might be suggestive of a change in risk aversion as specific to social trust.

### Relationship between adolescent development of trust and self-reported family adversity

Whilst in the earlier set of analyses we defined patterns of typical development in trust behaviours, our data also reveal that trust development is subject to substantial inter-individual variability (see, e.g. Fig. 2). Thus, we sought to examine whether self-reported family experiences, particularly parenting, might explain this variability. Building on previous work using the same dataset^28^, we repeated our analyses but now included a regressor indexing participants’ childhood family experience prominently including experiences with their parents (see methods for details of principal component analysis based on ref. ^28^), where higher scores denote a greater degree of self-reported family adversity.

We found no evidence for a main effect of adversity (F(1,506) = 2.04, *p* = 0.153, estimate = −0.05, se = 0.03), nor any statistically significant interaction of adversity with mean age (F(1,506.00) = 1.57, *p* = 0.211) on mean trust as indexed by round-by-round investment behaviour. However, we found a statistically significant interaction of adversity and longitudinal development on mean trust (F(1,9696.00) = 6.83, *p* = 0.009, estimate = −0.03, se = 0.01). This finding suggests that those reporting more positive family experiences showed a steeper growth of trust in terms of round-by-round investment with development (Fig. 3, S-Table 3). By contrast, those reporting more pronounced family adversity showed an attenuation in a growth of trust as they got older. There was no evidence for an interaction of self-reported family experiences and longitudinal development on round 1 investments (i.e., a-priori trust) (F(1,507) = 1.83, *p* = 0.177, estimate = −0.04, se = 0.03).

In a next step, we examined a computational basis for the influence of family adversity on trust behaviour and its development. We deployed a series of mixed models (7 models in total) with parameters from our computational model as dependent variables, and cross-sectional age, longitudinal age, family experiences as well as all 2-way interactions as predictors. Employing a Bonferroni-corrected threshold (for *n* = 7 parameters, 0.05/7 = 0.007) test of significance the sole effect of family experience related to a main effect on the Irritability parameter (F(1506) = 7.92, *p* = 0.005, estimate = 0.006, se = 0.002). As can be seen in Fig. 4, greater family adversity was associated with higher Irritability estimates, across both measurement time points. Higher values on this computational parameter index a propensity to enter an irritated state, wherein participants are disposed to punish their partner upon experiencing below-expectation trustee returns (retaliation). Importantly, as operationalized in our computational model, this retaliation occurs in a state of ‘mentalization breakdown’^34^, characterized by absence of planning, no Theory of Mind and no aversion to oneself or the partner losing money.

To map our computational modelling parameter Irritability onto investment behaviour, we conducted a proof-of-principle analysis on a behavioural marker of retaliation (i.e., a reduction of investment after an unfair trustee action) enabling us to ascertain if this is affected by reported family adversity (see SI results for details). Paralleling our modelling results, this revealed a statistically significant effect of family adversity on the degree of retaliation F(1,540.15) = 3.98, *p* = 0.047, estimate = −0.06, se = 0.03, see S-Fig. 6B). Note that whilst Irritability related to family adversity per se, independent from development, there was an uncorrected effect for an interaction of family experience x longitudinal development on the developmentally relevant social risk aversion parameter (F(1,507) = 4.44, *p*_uncorrected_ = 0.036, i.e., paralleling the significant interaction effect seen in the analysis of investment behaviour, but not surviving Bonferroni correction.).

In a next analysis step, we were interested in elucidating the relationship of trust, family adversity and the longitudinal development of peer relations.

In our sample, quality of peer relationships was longitudinally assessed at 3 measurement time points (T1_q_: around T1 of the task measurement, T2_q_: mean 1.13 (SD = 0.30) years after T1, T3_q_: 2.26 (SD = 0.22) after T1_q_) using the Cambridge Friendship Questionnaire (CFQ, a measure independent of parenting^28,29,35^ see Methods). This sampling enabled us to examine whether the development of trust predicts social relations later in adolescence, including establishing a capacity to form, and maintain, adaptive social relationships^1^, in a period of life when individuals need to navigate social environments with greater independence. Thus, our longitudinal approach allowed us to test a hypothesis of whether trust, as measured in the laboratory via a standardized economic game, predicts the development of future social relationships in the longer term, and whether this is moderated by family experiences. Better family experience scores were associated with higher CFQ scores at T1_q_, but also predicted peer relation quality at the latest longitudinal time follow-up time point, ~2.3 years later (T1_q_: t(377) = −5.863, *r* = −0.289, *p* < 0.001, T3_q_: t(377) = −3.305, *r* = −0.168, *p* = 0.001). Thus, those with adverse past family experiences reported a lower satisfaction with their current peer relations, and self-report of past family adversity predicted lower satisfaction with future peer relations. This finding lends evidence to the general notion that social experiences across different developmental periods are interdependent, with past experiences impacting successive stages^23^.

**Fig. 5:**
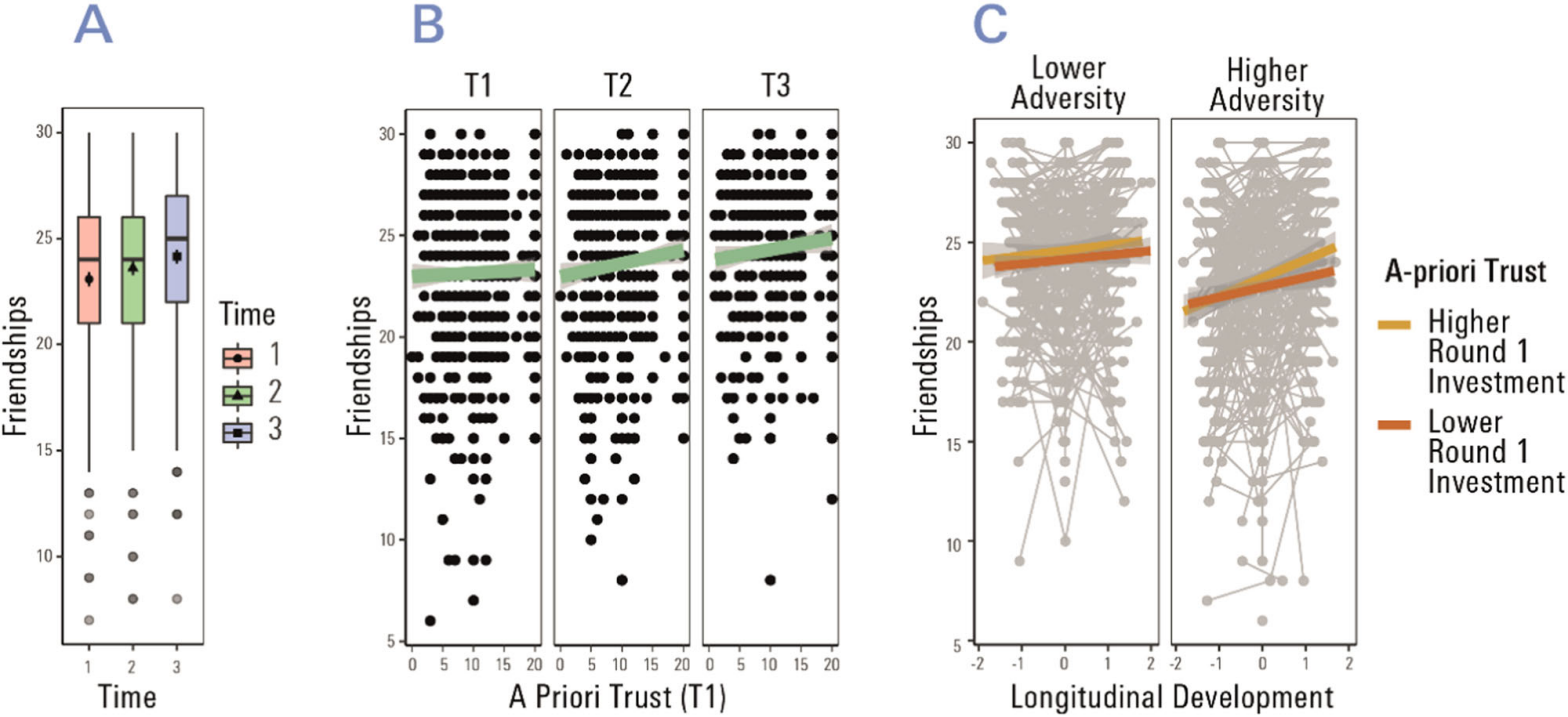
Developmental trajectories of peer relation quality and their association with a-priori trust and self-reported family adversity. **A** The quality of peer relations (measured by self-report via the CFQ) increased from measurement time point 1 until 3. We plot posterior estimates and model-based errors (confidence intervals) from the mixed model based on *n* = 786 participants, using the function afex_plot (R package afex)^69^. The box in the boxplot represents the middle 50% of the data, the line drawn through the box represents the median, the symbols represent the mean. The lower and upper edges of the box represent the first and third quartiles, respectively. **B** A priori trust at baseline (i.e., investments in round 1 of the multi-round trust task at T1) was positively correlated with peer relation quality (statistically significant main effect of T1 round 1 investment on friendships across all measurement time points). **C** Significant interaction of a-priori trust, longitudinal age and family experiences. Those with higher self-reported family adversity who showed a higher degree of a priori trust at baseline reported the strongest gain in peer relation quality (measured by the CFQ) over the longitudinal follow-up period. Longitudinal age was calculated with respect to age when the CFQ was completed (T1_q_, T2_q,_ T3_q_). The error band represents the standard error of the mean at each level of a priori trust.

We next asked whether our trust measures at T1 (baseline) are predictive of the long-term development of the quality of peer relationships (T1_q_ → T2_q_ → T3_q_), and whether this longitudinal association is moderated by family adversity. The quality of friendships increased significantly with measurement time points (F(2,1221.62) = 24.70, *p* < 0.001, contrast estimate(T1_q_ → T3_q_) = 1.06, se = 0.15, Fig. 5A). We found participants who showed more a priori trust at T1 (in terms of round 1 investments in our task) reported more pronounced positive peer relation quality (F(1,757.74) = 5.27, *p* = 0.022, estimate = 0.06, se = 0.02; Fig. 5B, effect was not statistically significant for mean investments, F(1,759.72) = 3.10, *p* = 0.069, estimate = 0.044, se = 0.014). This indicates that initial (i.e., unconditional) trust is associated with establishing and maintaining high-quality real-life social relationships. The latter is indeed a central developmental task in adolescence, a period of life where navigating the social world becomes independent.

In a final analysis step, we asked whether the effect of a-priori trust on the subsequent development of peer relations was moderated by family experiences. We observed a significant interaction of a priori trust and adversity with longitudinal development of friendships from T1_q_ over T2_q_ and T3_q_ (F(1,1050.70) = 6.77, *p* = 0.009, estimate = 0.05, se = 0.01; Fig. 5C, see S-Table 10 for full output of the model). As Fig. 5C shows, those reporting higher family adversity expressed a stronger positive association of baseline a priori trust with a gain in the quality of peer relations. Taken together, these observations are consistent with the notion that unconditional trust acts as a resilience factor in those with higher self-reported adversity, enabling the establishment of adaptive peer relations in the face of more negative past family experiences.

## Discussion

Leveraging a longitudinal sample of adolescents and young adults we detail typical developmental trajectories in trust behaviour, as well as inter-individual differences therein. Inter-individual differences in dimensions of adolescent trust development are associated with self-reported past family adversity and future peer integration.

First, we show that trust increases from adolescence into young adulthood. Previous cross-sectional studies, involving a comparable age range as our study (i.e., mid-adolescence to adulthood) have shown heterogeneous results, with some studies finding no evidence for age-related changes in trust behaviour^16,17^. Others, including our previously published cross-sectional analysis of this study’s baseline datasets, showed older participants manifest greater trust compared to younger participants^12,36^. Importantly, by disentangling cross-sectional age effects from longitudinal developmental effects, we extend upon these previous cross-sectional findings to show not only age-related, but also within-person, increases in trust behaviour.

Trust necessarily entails risk^11,37^ related to an uncertainty regarding whether trust behaviour will be reciprocated by the other. In the context of an economic game this includes a financial (earning less money in this trial) as well a social (e.g. a risk of being taken advantage of; loss of face) component. Our modelling analyses indicate that developmental changes in social risk preferences act as a key cognitive process contributing to an increase in trust across adolescence. Thus, social risk aversion decreased with age, and intriguingly, it also decreased intra-individually over a 1.5 year longitudinal follow-up period. Our data suggest that this developmental change was adaptive in so far as less social risk aversion on average lead to a higher total task pay-out. Put differently, in our sample younger participants subjectively valued money they kept more than money they invested and were repaid following a social interaction, even though this strategy entailed they earned less money. By contrast, within the same sample, cross-sectional data on non-social risk-taking (gambling) indicated the absence of age-related change in risk behaviour in the non-social domain. This speaks to a potential specificity of social risk development in adolescence, an interpretation in line with a proposal that adolescence is a period characterised by hypersensitivity to social risk^38–40^. It is argued that (prima facie) irrational behaviours (as observed in our task by less advantageous investment behaviour) might reflect attempts to avoid occasions of social risk. Thus, one interpretation is that, for younger adolescents, being taken advantage of by a given amount is more painful than an equivalent degree of generosity is rewarding.

Despite, on-average, a significant cross-sectional and longitudinal increase in trust across our sample, we show this developmental effect is subject to substantial inter-individual variability. A key hypothesis in developmental psychopathology research proposes that previous family experiences are a source of variability for later social development^23–27^. More specifically, experiences with primary caregivers are believed to provide a template for social behaviour that shapes later life interpersonal interactions^41^. It is plausible that this is relevant at an adolescent phase when individuals are confronted with  the developmental challenge of becoming more independent through the establishment of extra-familial social relationships. In line with this, we show that scores on a retrospective self-report family experience factor predicted the longitudinal development of trust behaviour from adolescence through to young adulthood. More specifically, those reporting higher family adversity involving more negative parenting had an attenuated longitudinal gain in trust, as indexed by their investments in our multi-round trust game. This accords with the notion that a secure family environment serves as a basis for adaptive trust later in life (i.e., in adolescence). It also complements cross-sectional evidence from clinical populations, who had experienced more severe forms of interpersonal trauma than was the case for a majority of the current sample, where in the trust game previous extreme adverse interpersonal circumstances is linked to reduced trusting behaviour ^42,43^.

Building on our previously validated computational model^11,12^, we identified a computational basis for the influence of family adversity on trust behaviour, evident in higher modelling-derived Irritability estimates. This parameter indexes a propensity to enter an irritated state, whereby participants are more disposed to punish a partner when experiencing below-expectation trustee pay-backs. Importantly, as operationalized in our computational model^10,11^, this type of retaliation occurs in a state of ‘mentalization breakdown’^34^ or a ‘hot emotional state’^11,44^. That is, irritability in our computational model is characterized by an absence of planning, forgoing potential gains from cooperation, a disregard for beliefs of the other player, as well as lack of guilt. Importantly, this form of retaliation differs from what might be called strategic retaliation, where the latter has an explicit aim to teach a lesson. These findings are consistent with the Social Information Processing Theory which describes how social information processes shape social adjustment in children and adolescents^45^. In line with this theory, adolescents who self-report higher levels of family adversity might perceive and interpret below-expectation trustee actions in a hostile way, thereby increasing a likelihood of retaliation rather than ignore the trustee’s action or try to repair trust. In a vicious circle type of spiral, in consequence, this might lead to less satisfying social interactions. Such a hostile attribution/interpretation bias of ambiguous social situations is suggested to develop as a consequence of maladaptive parenting^46^ or interpersonal trauma^47^.

It is interesting to consider that in this study, effects of early family experience played out in a task that involved an interaction with an unknown other. One interpretation is that effects of adversity unfold in novel social situations, where a previously learnt template of how much to trust might inform decision-making more strongly than is the case for interactions with close others with whom an agent has specific social learning experiences. An interesting future avenue of research might be to experimentally contrast trust towards familiar others (e.g. friends, partners, family) with trust towards strangers, as tested here in a young population with a history of family adversity. Indeed, in other social decision-making contexts, it has been shown that friends differentially bias computations about risk in adolescents^48^, and that adolescents show greater prosocial behaviour towards their friends^49^. At a clinical level, irritability in the face of mentalization breakdown is suggested as an important factor contributing to the development and maintenance of borderline personality disorder (BPD^50^), a condition often associated with adverse family experiences^51,52^ as well as altered trust behaviour in the trust game^7,11,53^.

Trust is considered a core ingredient for establishing and maintaining social relationships^54,55^, and here we show that a priori trust in our task (i.e. round 1 investment at measurement time point T1) is predictive of the development of the quality of peer relationships until 3 years later. This extends previous findings showing that trust beliefs (as assessed via a questionnaire) negatively predict longitudinal changes in loneliness and maladaptive peer interactions during childhood and young adulthood^56,57^. It also chimes with large-scale survey data showing a cross-sectional association of negative expectations about others and perceived loneliness^58^. Further, we show that the association of trust with future peer relations was driven primarily by those with more adverse family experiences. This is suggestive that interpersonal trust acts as a resilience factor, enabling the development of more satisfying independent relationships also when past social interactions within one’s own family are perceived as adverse.

### Limitations

By design we have relied on retrospective, self-reported family experiences^28^, and several biases are known to arise from retrospective self-reports of adverse experiences in the context of child maltreatment (See^59,60^ for discussion). However, previous work has found that negative childhood experiences are more likely to be under- as opposed to over-reported^61^, while the clinical relevance of self-report measures is emphasized by a link between self-reported childhood adversity and early adult psychopathology^59^. A further limitation is the fact that the majority of our sample reported low levels of negative family experiences and this might explain the modest effect sizes observed in our study, highlighting a need for large samples.

In conclusion, we show that trust increases from adolescence through to young adulthood, and that unconditional trust measured in the laboratory is predictive of a beneficial development in real-life peer relations 3 years later. We find evidence for an important role of past social family experiences on the evolution of trust, wherein those reporting higher family adversity had an attenuation in a gain of trust from adolescence through to young adulthood. Finally, our study showcases the power of large, multivariate, longitudinal studies in characterising the development of trust in adolescence^62^, a period of life where the consolidation of social relationships is a key developmental challenge.

## Methods

### Sample

The experimental task (Fig. 1A) was delivered as part of a task battery in a sample of community dwellers between the ages of 14 and 24 in Cambridgeshire and London, as part of the Neuroscience in Psychiatry Network (NSPN) project^63^. All participants, and where participants were underage their legal guardians, provided written informed consent. The Cambridge Central Research Ethics Committee approved the study (12/EE/0250). Participants were paid £10 for completing the Home Pack Questionnaires and up to £150 for participating in on-site assessments (MRI and/ or cognitive task battery), depending on which tasks they completed. Data for this task were available from *n* = 570 (285 female) participants for baseline and follow-up. When signing up for this study, participants were asked to tick: Sex: ‘Female’ or ‘Male’. It was not clarified if some understood the question as ‘gender identity’, socially attributed or biological category. Due to the phrasing ‘Sex:’ one might speculate that most participants understood the question to mean ‘self-reported estimate of biological sex’, which is why we use the term ‘sex’ in this article, even though we do not have disaggregated information on participants’ sex vs. gender.

As the data reported in this paper were acquired as part of a larger study including other cognitive tasks and MRI assessments^63^, no statistical method was used to predetermine sample size for this specific task, however sample size was chosen to exceed previous published studies on trust development. In general, the sample size for the NSPN cognitive task battery was as large as study resources allowed, including resources needed to re-contact participants, up to achieving a follow-up rate of at least 70%. No data were excluded from the analyses. Aspects of the baseline data, with a focus on computational modelling, have been reported previously^12^. Participants were 14.10–24.99 years old (mean = 19.05, sd = 2.96) at baseline. Note that we use the term adolescence for the whole age range. Mean age at follow-up was 20.30 years (range: 15.11–26.48 years, sd = 2.98). Mean time between first and second task assessment was 1.48 years (range: 0.99–2.6 years, sd = 0.29). Structural imaging and task data were available (and passed quality assessment) for *n* = 294 participants.

A subsample of *n* = 55 participants completed the task three times, with an additional interim session after a ~6-month follow-up period (see refs. ^29,30,64^ for this approach). This “retest sample” allows us to index short-term changes (over ~6 months), indicative of training effects, from long-term changes (over ~1.5 years) indicative of developmental change.

### Multi-round trust task

Participants engaged in an iterated 10-round trust game, similar to that used in previous studies of adult healthy and psychiatric populations^6^. The task was programmed in MATLAB 2012 with the Cogent 2000 toolbox. Trained research assistants provided instructions to the participants regarding the accurate rules of the game. This game was a part of a larger set of decision-making tasks (as detailed in Kiddle et al.^63^). Before the game started, participants were tested for their understanding of the rules and encouraged to ask any questions. All participants included in the study understood the task well, agreed to participate, and provided data that was of sufficient quality for analysis.

Participants took on the role of the “investor” throughout the game and were instructed to play this role according to their own goals and preferences. Participants were led to believe they were playing with an anonymous peer (the trustee) from the same study, playing from another site, who would also be paid in proportion to their own winnings. No information was provided about name, gender or background of the partner. Note that with this experimental approach, we explicitly tested trust in an unfamiliar social situation, a scenario where we hypothesized general templates about how much to trust would play out most prominently. Participants were informed that they would receive monetary rewards in proportion to their winnings. For details of the experiment, see Fig. 1.

In each of the ten rounds, the investor received an initial endowment of 20 play-coins, and then decided the amount (in whole coins) to transfer to a trustee. Participants knew that the amount invested would be tripled by the experimenter, before the trustee (in our case, unbeknownst to the participants, the computer algorithm) decided how many coins to return to the investor. The repayment by the trustee was not increased by the experimenter. After the trustee’s action, the investor was informed of the outcome, and the next round started. At the end of the study, participants were debriefed that the trustee had in fact been a computer algorithm that emulated the behaviour of healthy adult trustees based on previous studies. No randomization was used in this experiment.

### Non-social risk paradigm

Only at T1, participants underwent an additional roulette-type of risk-taking task, following closely the methodology described in^32^. The gambling task involved a choice between a sure amount and a four-sector roulette, defining an expectation, variance and skewness over roulette outcomes. See refs. ^32,33^ and S-Fig. 4 for more details.

### Self-reported family experiences

Our approach is based on a previous publication using the same dataset where we applied Principal Component Analysis (PCA) to the baseline measurements in order to derive a dimensional, composite measure of childhood family experiences^28^. In the NSPN cohort, such family experiences were assessed via two self-report measures to assess early life parenting behaviours: the Alabama Parenting Questionnaire (APQ) and the Measure of Parenting Styles (MOPS^65^). Whilst most of the subscales assess negative parenting experiences, two subscales of the APQ also indicate positive family experiences (subscales involvement and positive parenting^28^, see Supplementary methods). Questionnaire data were available for *n* = 511 participants of the sample, assessed via a sending paper-pencil questionnaires to their home address. To calculate that family experience factor, we used the baseline questionnaire assessment (which took place at mean = 0.43 years, sd = 0.35 years before the T1 task assessment). See the Supplementary methods as well as the previous publication^28^ for details on the used questionnaires.

#### Principal component analysis

We calculated a multi-modal composite score for family adversity as described in a previous publication on the same dataset^28^. We conducted a PCA on standard-normally transformed individual total scores of the MOPS the APQ subscales using the R Package *Lavaan*^66^. From both analyses, we extracted individual scores for the first component to reflect retrospectively recalled childhood family experience scores.

### Quality of peer relations

To evaluate the development of peer relation from baseline to follow up, we used the Cambridge Friendship Questionnaire (CFQ^28,29,35^ freely available at https://osf.io/cf59r/), measured three times as part of a questionnaire battery around the time of the behavioural task assessment (baseline), as well as at a median of 1 (T2_q_) and 2.3 years later (T3_q_). In a previous publication, this measure has been shown to be longitudinally linked to psycho-social resilience in this sample^28^. See SI for details.

### Statistical analyses

All data were analysed using R (Version 4.0.3) and Rstudio (Version 1.1.383)^67,68^. Mixed models for cross-sectional and longitudinal analyses of trust and longitudinal analyses on reciprocity and parameters derived from our computational model were fit using the R package *afex(Version 0.28.1)*^69^. *P*-values were calculated based on a Sattherthwaite approximation for degrees-of-freedom. All statistical tests were two-tailed. Subject-specific slopes were estimated as random effects. We additionally fit ordinal mixed models using the R package *ordinal*^70^ to check for potentially deviating results due to the scaling of the computational parameters as outcome variables (Investor Irritability, Belief about Trustee’s Irritability). This did not indicate any qualitative deviation from the linear mixed model (see S-Tables 5 and 7 for results of the ordinal mixed models). In all mixed models, sex was included as a covariate, due to previous findings on sex differences in trust development in adolescence^12,15,16^, (see S-Fig. 1). Additionally, based on findings in the T1 dataset^12^ we ran control analyses for all developmental mixed models including baseline IQ and socioeconomic status, results of which are reported in the Supplementary Note 1, and which did not lead to substantial deviations of results reported in the main manuscript. All continuous predictors were centred on zero. Effect-coding was used for contrasts. Post-hoc contrasts were computed using the R package *emmeans (Version 1.6.0)*^71^. Plots were generated using *ggplot2 (Version 3.3.3)*^72^.

#### Longitudinal mixed model

First, to disentangle longitudinal development from age effects, participants’ age at the time where they completed our task was separated into within-subject (longitudinal) and between‐subject (cross‐sectional) components (as recommended in^73,74^). Specifically, “cross‐sectional age” for a participant *i* was calculated as age_i_–mean age (where age_i_ is participant *i*’s mean age across visits and mean age is the mean age across the whole sample). Thus cross-sectional age indexes a participant’s mean centred age with respect to the sample’s age. Longitudinal age for participant *i* at time point *j* was calculated as age_ij_–mean(age_i_), where mean (age_i_) is a participant’s mean age across the two measurement time points. Thus, “longitudinal age” reflects the within‐subject centred deviation from the participant’s own mean age. This distinction allows an assessment of true within‐subject change (taking into account the temporal distance between two measurements), independent of age effects:

$${{{{{\rm{Behavioural}}}}}}\; {{{{{\rm{Task}}}}}}\; {{{{{\rm{Measure}}}}}} \sim {{{{{\rm{age}}}}}}\; {{{{{\rm{longitudinal}}}}}}+{{{{{\rm{age}}}}}}\; {{{{{\rm{cross}}}}}}{\mbox{-}}{{{{{\rm{sectional}}}}}} \\+{{{{{\rm{age}}}}}}\; {{{{{\rm{longitudinal}}}}}}\times {{{{{\rm{age}}}}}}\; {{{{{\rm{cross}}}}}}{\mbox{-}}{{{{{\rm{sectional}}}}}}+{{{{{\rm{sex}}}}}}+(1{{{{{\rm{|Participant}}}}}})$$

For the analysis of round-by-round investments, trial number^1–10^ was included as an additional regressor. Note that mirroring the results of the previously published cross-sectional analysis, we did not observe any statistically significant quadratic effects of age (cross-sectional age²) or a significant interaction of cross-sectional age² and longitudinal age in our mixed models predicting i) trial-by-trial investments or ii) investment in trial 1 (all Fs < 0.21, all ps > 0.65), which is why quadratic age effects were not considered in all further analyses of inter-individual differences.

To analyse the association of family experiences with trust, the factor-analytically derived family experience score (as well as 2- and 3-way interactions) was added as an additional regressor.

To analyse how baseline trust (measured at T1) influences the longitudinal development of self-reported peer relation quality, we predicted scores on the Cambridge Friendship Questionnaire (CFQ, baseline (T1 _q_) and 2 follow-up (T2_q_, T3_q_) measurements) with investments at T1, longitudinal and cross-sectional age at the time of questionnaire assessment (as well as their 2- and 3-way interactions) and gender as a covariate in mixed effects regression models. In a follow-up model, we added the family experience factor as a moderator. Note that in this analysis, we had more participants available, as we could include all participants who had CFQ data as well as T1 trust task data available (i.e. those that dropped out for the second task measurement could be included). Note that as this analysis used CFQ data as a dependent variable, longitudinal/cross-sectional age (decomposed as described above) referred to the age of the participant when the CFQ questionnaires were completed in this analysis (T1_q_, T2_q_, T3_q_).

We used mixed effects models as they have been shown to be robust towards violations of distributional assumptions^75^.

### Computational modelling

#### Computational multi round trust task model

We model the participant’s behaviour as an interactive partially observable Markov decision process (I-POMDP, see ref. ^76^. This is a class of decision making models that includes a model of tactical depth of thinking, when performing a task with other agents. Thus, it provides a way of modelling what an agent thinks the other agents are thinking and how this will influence choices.

An I-POMDP consists of a set of possible states and beliefs (in our case defined by how trustworthy the partner appears, how sophisticated a participant’s model of their partner is, whether participants act as investor or trustee and how close the end of the game is), possible actions (in our case, investment and repayment options) and possible observations (in our case, the choices made by the social partner). Furthermore, an I-POMDP contains a set of transition probabilities, determining the likelihood of ending up in a new state after choosing an action and a set of observation probabilities, encoding the probabilities of making a given observation during a state transition. In the case of the multi round trust task, the transition and observation probabilities consist of the likelihoods of partner choices, based on the observed game history so far^10^. During the interactions, information about the partner is encoded through Bayesian updating of their trustworthiness (their inequality aversion type, see below), based on choice probabilities under certain parameters. In the case of the MRT model, there are 7 parameters, which determine the generative model of choices in the game, forming a vector *ϴ* = $(\alpha,\, \omega,\, k,\, P,\, \zeta,\, q\left(\zeta \right),\, \beta )$. See Table 1 for a conceptual description of all parameters.

In the following, we will go through the parameters of the model validated in^11^ and detail how they influence the interaction. Note that our estimation operates in a space of game trajectories rather than just the space of 10 investor’s choices. Since reputation building and planning ahead are essential and all previous trials influence the belief state of the participants, we operate in a space of 21 (investor-trustee choices per round) to the power of 10 possible game trajectories. Further methodical detail can be found in^10,11^.

First, in each round, payoffs for the investor ${\chi }_{I}$ and the trustee ${\chi }_{T}$ are achieved based on the investor action (investment) ${a}_{I}$ and the trustee response (repayment) ${a}_{T}$:

1 $${\chi }_{I}=\left(20-{a}_{I}\right)+{a}_{T}$$

2 $${\chi }_{T}=3{a}_{I}-{a}_{T}$$

Following earlier work^77^, for computational reasons, the action space of the investor is discretized to $\left\{\left[{{{{\mathrm{0,2}}}}}\right],\left[{{{{\mathrm{3,7}}}}}\right],\left[{{{{\mathrm{8,12}}}}}\right],\left[{{{{\mathrm{13,17}}}}}\right],[{{{{\mathrm{18,20}}}}}]\right\}$ (with representatives 0, 5, 10, 15 or 20). The trustee response conditional on that is {0, 1/6, 1/3, 1/2 or 2/3) of the amount invested by the investor and tripled by the experimenter, leaving both investor and trustee with 5 possible actions, if the investor invests more than 0.

##### Inequality aversion

We adapt the concept of an inequality aversion utility introduced by ref. ^78^ to evaluate the utility ${u}_{I}$ of an inequality averse investor with parameter α ≥ 0 to

3 $${u}_{I}={\chi }_{I}-{{{{{\rm{\alpha }}}}}}\max \left\{{\chi }_{I}-{\chi }_{T},\, 0\right\}$$

And an inequality averse trustee to

4 $${u}_{T}={\chi }_{T}-{{{{{\rm{\alpha }}}}}}\max \left\{{\chi }_{T}-{\chi }_{I},\, 0\right\}.$$

It follows from this formula that an inequality averse (α = 1) trustee will seek to repay an investor to the point where they both have equitable outcomes. Such a trustee can be considered trustworthy (meaning they will reciprocate reliably) and the goal of an investor’s beliefs and inferences in the MRT can be modelled as to be learning whether their partner falls into this category. Conversely, an inequality averse investor will invest at least a minimal amount in the trustee, opening up some possibility of the trustee to exploit them (since such an investor would keep investing, even if they discovered the trustee to be a poor reciprocator).

For computational reasons and also since the generated behavioural profiles do not change drastically as a function of small differences in inequality aversion^10^. we use a discrete set comprised of 3 possible values of inequality aversion *α* *∈* {0,0.4,1} and we assume agents to learn about the partner’s inequality aversion setting, using a Dirichlet prior on a categorical distribution on the 3 values, with initial Dirichlet prior settings being $\left({A}_{0},{A}_{0.4},{A}_{1}\right)=({{{{\mathrm{1,1,1}}}}})$. During the interaction, our agents were modelled to update according to an approximate rule, using the probability $p({o|}{{{{{\rm{\alpha }}}}}})$ of the partner choosing an action *o* given each respective inequality aversion value:

5 $${A}_{{{{{{\rm{\alpha }}}}}}}^{{new}}={A}_{{{{{{\rm{\alpha }}}}}}}^{{old}}+p({{{{{\rm{o}}}}}}|{{{{{\rm{\alpha }}}}}}).$$

Furthermore, we specify that, since an investment of 5 yields no effect of inequality aversion (as both partners end up with an amount of 15 following the investor action), we only investors update their beliefs on trustee responses to investments greater than 5.

##### Risk aversion

The investor’s utility is further modified by a model parameter denoting investor’s risk aversion ω. This encodes a (investor) preference for money kept (and thus securely obtained) over money given in the final form of the investor utility ${{u}_{I}}^{{final}}$:

6 $${{u}_{I}}^{{final}}={{{{{\rm{\omega }}}}}}\left(20-{a}_{I}\right)+{a}_{T}-{{{{{\rm{\alpha }}}}}}\max \left\{{\chi }_{I}-{\chi }_{T},\, 0\right\}.$$

Risk aversion was discretized to levels of ${{{{{\rm{\omega }}}}}}\in$ {0.4, 0.6, 0.8, 1.0, 1.2, 1.4, 1.6, 1.8}.

The trustee has no uncertainty about the amount they will be receiving each round, but they do have an analogue parameter ${b}^{T}\left({{{{{\rm{\omega }}}}}}\right)$, encoding their assumption on the investor’s risk aversion, as a static non-updated value, to be used in their planning.

##### Planning

The structure of the multi-round trust task is such that planning is an important part of the game dynamics^10,77,79^. The planning parameter *P* encodes the number of future interactions that are taken into account when making a decision. A planning value of *P* = 1 encodes an agent which takes into account the effect of the current action on the expected payoffs in the next 1 rounds as well. *P* was considered for values of $P\in \left\{{{{{\mathrm{1,2,3,4}}}}}\right\}$, since our model validation studies showed that beyond these values, behavioural preferences appear not to vary much anymore, and depth of planning is one of the most computationally costly parameters^10^.

##### Theory of mind

The tactical depth of thinking (i.e. the model of how the agent thought their partner was reasoning about them, see also^80,81^ was encoded in a separate parameter $k\in \left\{{{{{\mathrm{0,1,2,3,4}}}}}\right\}$, also called “theory of mind level” (ToM). A level 0 agent holds static (non-learning) models of their partners (dubbed “level −1”) and updates their beliefs about the partner based on those. A level 1 agent models the partner as level 0 (i.e. they are aware the partner is learning about them) and updates their own beliefs based on these models. In general, a level k agent models their partner as level k-1 and updates their beliefs based on a set of level k-1 models for different possible parameter values. We already mentioned our agents are learning about their partner’s inequality aversion (and therefore implicit “trustworthiness”). As described in^11^, they are also assumed to learn about the partner’s propensity to retaliate harshly (their “irritability”).

##### Irritability

Irritability *ζ* is a parameter with levels *ζ* ∈  0;0.25;0.5;0.75;1 that governs a shift from a rational or non-irritated state to an irritated state. If an agent’s investment or repayment is below what their partner expects (i.e. below the expected value calculated based on partner models) and the partner is irritable (*ζ* > 0), then the decision making policy of the irritable partner is shifted to a mixture policy (with w(*ζ*) = *ζ*)

7 $${\mathbb{P}}\left[a\right]=\left(1-w(\zeta )\right){\mathbb{P}}\left[{a|nonirritated}\right]+w(\zeta ){\mathbb{P}}\left[{a|irritated}\right].$$

Here, the irritated choice probability is characterized by a planning horizon of 0 (no planning), an inequality aversion of 0 (no concern for fairness), a ToM level of −1 (no concern for partner beliefs) and a risk aversion of at least 1 (if the original risk aversion was below one), with the other parameters remaining the same.

If an already irritated agent becomes again irritated, their policy shifts even further (additively) towards the irritated policy ($w\left(\zeta \right)=w\left(\zeta \right)+\zeta$), with a maximum of 1 ($w\left(\zeta \right)=1$, fully irritated). If, conversely, an investment or repayment above expectations is observed by the irritated agent, they shift back to the non-irritated policy at the same rate ($w\left(\zeta \right)=w\left(\zeta \right)-\zeta$).

##### Irritation awareness

Being aware that partners may be irritable changes behaviour in the MRT considerably (agents try not to irritate partners, see Hula et al, 2018), therefore we model our agents as holding a belief about the partner’s irritability and learning more during the interaction. We model the probabilities for different irritability settings as a categorical distribution, with a Dirichlet prior on it. For updating the Dirichlet prior, we use the same approximate updating rule as above. Unlike for inequality aversion, where all agents are assumed to start with a uniform prior, there exist five starting values for the irritability settings, which form the final parameter of the model, the irritability awareness $q({{{{{\rm{\zeta }}}}}})\in \left\{{{{{\mathrm{0,1,2,3,4}}}}}\right\}$. The five settings correspond to being “irritation ignorant”, “irritation unlikely”, “irritation possible”, “irritation likely“ and “irritation certain” (see details in)^11^.

##### Choice stochasticity

How agents make choices in the trust task (choice making probability ${\mathbb{P}}{\mathbb{[a]}}$) is assumed to rely on a logistic softmax function, based on the expected utility $Q(a)$ of a current action (with the utility of future actions being obtained from a Bellman-equation mechanism)^10,79^

8 $${\mathbb{P}}[a]=\frac{{e}^{{{{{{\rm{\beta }}}}}}{Q}(a)}}{{\sum }_{c}{e}^{{{{{{\rm{\beta }}}}}}{Q}(c)}}.$$

In this equation, the parameter $\beta \in \left\{\frac{1}{4},\frac{1}{3},\frac{1}{2},\frac{1}{1}\right\}$ is the temperature parameter, which encodes how stochastic the choice preferences of an agent are (lower values encoding higher stochasticity).

To estimate individual parameters, we employed a full search over the parameter space and used the combination of parameter values that produced the smallest negative loglikelihood of generating the observed sequence of choices, given the game’s history of actions and responses. The parameters were kept on a discrete grid, for computational reasons (explicit coding of the beliefs and inferences in particular), a limitation that ongoing work is aiming to lift in the future. The parameter grids were determined from simulations and plausibility consideration (see explanations above as well as refs. ^10–12^. Table 1 shows an overview including parameter ranges and a conceptual description of each free parameter used for the computational modelling analysis.

#### Model selection

To validate our modelling approach, we also compared the full model as described above (M1) with reduced variants of this model within the largest available dataset (T1 baseline dataset). To do so, we constructed reduced models as follows: a model with irritability = 0 and irritation belief = 0 (M2), a model in which, additionally to fixing irritability and irritation belief as in M2, also risk aversion and stochasticity were fixed (risk aversion = 1, softmax = 1/3, compare)^10^ (M3), and finally, a model which included all reductions of M3 and additionally set Theory of Mind = 0 (M4). We additionally include a full random model (M5) in the model comparison set. See^11^ for a more detailed description of this model selection approach for the models and task at hand.

As in previous work^11^ we evaluate the (average per model) negative log likelihood and use a Draper Bayesian Information Criterion (BIC)^82^ to compare models:

9 $${{{{{\rm{BIC}}}}}}={{{{{\rm{NLL}}}}}}+\frac{{{{{{\rm{p}}}}}}}{2} \,*\,(\log \left(10\right)-\log \left(2 \,*\, {{{{{\rm{pi}}}}}}\right))$$

where p denotes the number of parameters and 10 represents the 10 investor and trustee actions. As shown in Table 2, the BIC score indicated that the winning model was the full model M1 (7 parameters) as described above.

**Table 2:** Model Comparison Results

Model	Average BIC
M1 (Full model)	**26.56**
M2	28.33
M3	29.74
M4	30.5
M5	32.19

M1: full model as described in the Methods section, M2: model with irritability = 0 and irritability belief = 0, M3: model with additionally risk aversion = 1 and stochasticity = 1/3, M4: model with additionally Theory of Mind = 0, M5: random model.

#### Simulation of investment behaviour and posterior predictive checks

Based on our generative model and the individual parameter set derived for each participant, we simulated investment choices per participant and round. In a next step, we used this synthetic data to test for age, developmental and family experience effects based on the same mixed effects model we had used to analyse the empirical data. This reproduced the empirically observed findings very well (see Supplementary Note 1 and S-Fig. 5 and 6 for details).

### Brain behaviour co-development

See Supplementary methods, Supplementary note 1, and Supplementary discussion for a Region-of-Interest based analysis of the co-development of grey matter volume and parameters of trusting behaviour in a subset of our sample.

### Reporting summary

Further information on research design is available in the Nature Portfolio Reporting Summary linked to this article.

### Supplementary information

Supplementary Information

Reporting Summary

## Data Availability

Anonymized raw choice data and scripts generated for this study are available via the Open Science Framework https://osf.io/42b68/, 10.17605/OSF.IO/42B68. As noted above in more detail, for this study we re-use data from the NSPN project which was previously published^12,28,74^ and is also available via NSPNopen https://portal.ide-cam.org.uk/overview/6.

## Code availability

Custom code for the computational trust model and its validation is available here: https://github.com/AndreasHula/TrustGameCPSY and https://github.com/AndreasHula/BacktestingTrustNSPNBaseline; 10.5281/zenodo.8108911.
