# Supplementary material for: Self-reported childhood family adversity is linked to an attenuated gain of trust during adolescence
Source: Nat Commun. 2023 Oct 30;14:6920. doi: 10.1038/s41467-023-41531-z (PMC10616102; doi:10.1038/s41467-023-41531-z)
Supplement: Supplementary file 1 — Supplementary Information [file 41467_2023_41531_MOESM1_ESM.pdf]

Supplementary Information

Supplementary Methods

Reciprocity measures

Based on previous work [1] investor reciprocity on round j was quantified as $\Delta I_j - \Delta R_{j-1}$, where ΔI_j is the fractional change in investment from round $j - 1$ to j and ΔR_{j-1} is the last fractional change in repayment, relative to the money available to the trustee in this round (i.e., $(R_{j-1}/(I_{j-1} \text{ x } 3)\text{- } R_{j-2}/(I_{j-2} \text{ x } 3))$

Thus, positive values denote *benevolent* reciprocity, i.e. investors being generous (sending more) in response to a defection by the trustee. Conversely, negative values denote *malevolent* reciprocity, the investor reacts to the trustee's generosity with a breach of trust. Considering all investments together, decisions on average were benevolent in this sample (two-sided t-test against zero, t(701)=7.07, p<.001). Similar to the analysis of investments, investor's trial-by-trial reciprocity values entered a mixed effects model as a dependent variable with longitudinal age, cross-sectional age and Sex as predictors.

Based on [2], we additional operationalized 4 different strategies in terms of reciprocity (trust honouring, trust disrupting, trust repairing, distrust reciprocating) as the four quadrants of a circle as given by ΔI and ΔR. We then calculated both the radius and the polar angle of this circle, where the polar angle φ, denotes the direction of the investor's reaction (dependent on the trustee's action; where positive values denote a higher degree of cooperation), and the radius r denotes the magnitude of the investor's reaction.

	ΔR_{j-1}	ΔR_{j-1}
ΔI_j	Trust honouring	Trust repairing
ΔI_j	Trust disrupting	Distrust reciprocating (Retaliation)

Details about Questionnaires to assess Family Experiences and Quality of Friendships

Measure of Parenting Style: The MOPS is a self-report measure that assesses perceived parenting styles across three domains; indifference, over-control and abuse. Participants were asked to rate both their mother's and father's parenting behaviour on 15 statements, on a 4-point scale ('not true at all', 'slightly true', 'moderately true', 'extremely true'). The 'abuse' scale consisted of five items, asking whether maternal/paternal behaviours were verbally abusive, unpredictable, physically violent, elicited feelings of danger or elicited feelings of lack of safety. The 'overly controlling' scale consisted of four

items where maternal/paternal behaviour was overprotective, over controlling, critical, or made the participant feel guilty. Finally, the ‘indifference’ scale assessed six items of maternal/paternal behaviour where the parent was ‘ignoring, uncaring, rejecting, uninterested in, would forget about, or would leave the participant on his/her own a lot. Sum scores to responses in these items were calculated, with higher scores representing more abusive, over controlling or indifferent behaviour reported. Internal consistency was good for the maternal subscales (Cronbach's alpha maternal over control = 0.70, indifference = 0.86, abuse = 0.78). For paternal parenting, the internal consistency at baseline ranged from acceptable (Cronbach's alpha paternal over control = 0.65) to excellent (Cronbach's alphas paternal abuse = 0.88, paternal indifference = 0.93).

Alabama Parenting Questionnaire: The APQ [3] measures parenting practices. In NSPN, the nine-item short-form[4] was used and the ‘Corporal Punishment’ (three items) and ‘Involvement’ scale (three items) were added. Participants were asked to rate how typical each item occurred or used to occur in their family home on a 5-point scale ranging from ‘*never*’, ‘*almost never*’, ‘*sometimes*’, ‘*often*’ *to* ‘*always*’. We calculated sum scores for the five subscales: Positive Parenting, Inconsistent Discipline, Poor Supervision, Involvement, and Corporal Punishment, with higher scores reflecting higher frequency of the behaviour. Thus, high scores can indicate positive parenting (i.e. involvement, positive parenting) or negative parenting (i.e. inconsistent discipline, poor supervision, corporal punishment). Internal consistency at baseline was acceptable (inconsistent discipline & poor supervision: Cronbach's alpha > 0.62) and good (positive parenting, involvement, Corporal Punishment Cronbach's alpha > 0.71). Note that all results remained when the positive parenting scores (APQ positive parenting and APQ involvement) were removed from the analyses.

Questionnaire to assess perceived quality of peer relations / friendships

We used the Cambridge Friendship Questionnaire (CFQ) to assess the perceived quality of peer relations [5], a measure available as part of a Home Questionnaire Pack delivered close in time to the in-lab measurements [6]. The CFQ assesses the number, and quality of friendships via self-report (e.g. “How often do you arrange to see friends other than at school, college or work?”, “Do you feel that your friends understand you”, “Can you confide in your friends”). Higher scores signify higher satisfaction with peer relations. This measure has been shown to predict psycho-social resilience in this sample [5].

MRI Acquisition, Processing and Analysis

Acquisition and processing. Participants were scanned on three identical Siemens Magnetom TIM Trio whole-body 3T MRI scanners in Cambridge and London using the multi-echo FLASH MPM protocol [7] N= 193 subjects of our sample underwent structural imaging twice, for n=101 only a baseline structural scan was available.

Acquisition parameters were identical across sites. Whole-brain multi-echo FLASH MT weighted contrast were acquired at 1 mm isotropic resolution (TR: 23.7, α = 6, 176 sagittal slices, FOV = 256 × 240 mm2, matrix = 256 × 240 × 176). Semiquantitative MT saturation maps were derived using biophysical models[8{Tabelow, 2019 #5415}] using the hMRI toolbox (www.hmri. info) for SPM (Wellcome Centre for Human Neuroimaging, London, UK, http://www.fil.ion.ucl.ac.uk/spm). Whole-brain MT maps for all subjects were then segmented into grey matter (GM), white matter (WM), and cerebrospinal fluid, normalised to MNI space using geodesic shooting [9], and modulated to account for effects of normalisation using the Computational Anatomy Toolbox (http://www.neuro.uni-jena.de/cat/). Modulated grey matter maps thus reflect voxel-wise grey matter volume (GMV). GMV maps were finally spatially smoothed preserving GM/WM tissue boundaries [10] using a smoothing kernel of 6mm FWHM.

ROI Analysis

A recent coordinate-based meta-analysis [11], based on 23 original publications reporting on fMRI during economic trust games, found that decisions to trust most consistently involved anterior insula (AI). Based on this and further meta-analytic evidence on the role of the anterior insula in trusting behaviours [12] and risk processing [13], we hypothesized that anterior insula also plays important roles in the structural neuro-development of trust. Thus, mean grey matter volume (GMV) was extracted from the peak coordinates found in the meta-analysis (right AI MNI 44, 2, -42). These grey matter volume values were then entered into a mixed model as dependent variable, and predicted by social risk aversion (one regressor indexing cross-sectional (between-subject differences) and longitudinal (within-subject change) variance, respectively), as well as cross-sectional and longitudinal age. Additionally, two-way interactions of mean ("cross-sectional") social risk-aversion and both age components were included as predictors (please see [14,15] for a similar analytical approach in the same dataset). The same model was subsequently set up for the Irritability parameter, additionally including family adversity, following up on our behavioural results.

Movement, Sex, ethnicity, total intracranial volume and scanning site were included as nuisance regressors resulting in the following model specification for the ROI analysis:

```
Right AI Grey Matter Volume ROI ~ model parameter longitudinal + model parameter cross-sectional + age longitudinal + age cross-sectional + model parameter cross-sectional x age longitudinal + model parameter cross-sectional x age cross-sectional + movement + Sex + ethnicity + tiv + scanning site + (1|Participant)
```

where model parameter corresponds to the computational parameter of interest for following up our behavioural analyses (risk aversion, irritability).

To maximise sample size and thus power, all subjects undergoing MRI, including those that were scanned only at baseline were included in the MRI analysis, coding longitudinal age as “0” and cross-sectional age as the mean-centred age at T1 (compare [14] [15] for a similar approach in the same dataset).

Supplementary Note 1

Longitudinal Modelling of additional reciprocity measures (φ, r)

Cross-sectional age predicted the direction (φ) of investor’s responses to trustee’s re-payments (F(1,566.00)=10.04, p=.002), with participants who were older on average responding in a more cooperative manner. There was no significant effect of longitudinal development on the magnitude r nor direction (angle φ) of investor’s responses to trustee’s actions (Fs<1.60, p>.20).

Retest Subsample - Testing for training vs. developmental effects in a 6-month follow up subsample of participants

To analyse whether the observed longitudinal differences were predominantly due to retest effects or development, we included a sample of participants who were also tested 6 months apart (judged to be a short time with respect to maturation, henceforth 'short follow-up'). This comprised a sub-sample of n=55 of the total group who were tested three times in total (baseline, 6-month 'short' follow-up, 1.5-years 'long' follow-up), in the same manner as per our main sample (see Main Methods). This allowed us to differentiate retest effects from developmental effects for our key analyses of interest.

Round-by-round investment behaviour across the whole game. Testing for an effect of the factor time point (baseline, short follow-up, long follow-up) on round-by-round investments in the subsample that had completed all three measurements, revealed a significant effect of time point (F(2,1418.00)=4.77, p=.009). Critically, post-hoc analyses showed that investment behaviour increased significantly over the 18-month period (t=2.74, p=.006), and from the 6-month to the 18-month period (t=2.61, p=.009) but

importantly did not do so significantly over the 6-month period from baseline (t=0.13, p=.90). This pattern does not support a mere training effect, as if this was the case we would expect a stronger change after 6 months than after 18 months. See S-Figure 3.

Social Risk Aversion. Next, we analysed the effect of the factor time point (baseline, short follow-up, long follow-up) on social risk aversion in the short follow-up sample. We again observed a significant effect of measurement time point (F(2,106)=4.11, p=.02, S-Figure 3). Post-hoc analyses revealed that social risk aversion decreased significantly over the 18-month period (t=2.86, p=.005), but not significantly during the shorter periods (no significant effect from baseline to 6month follow-up (t=1.23, p=0.22), and from the 6-month to the 18 month follow up (t(106)=1.63, p=.11)).

Adjusting for IQ and socio-economic status as covariates when analyzing investment behaviour

In a set of control analyses, we included IQ at baseline to adjust for its effect on investment behaviour which we had reported in our previous publication [16]. IQ was positively associated with investments both in trial 1 (1, 566.00=78.61, p<.001), as well as across the whole game F(1,566.00)=79.21, p<.001). Reassuringly, the inclusion iof IQ did not change the observed effects of cross-sectional age and longitudinal age on investment behaviour (all ps remained <.001).

In a further set of control analyses, we included socio-economic status (if available at all) at baseline to adjust for its effect on investment behaviour which we had reported in our previous publication. Lower socioeconomic status was associated with less investments both in trial 1 (F(1,382=5.22, p=.023)), as well as across the whole game (F(1,382.00=9.21, p=.002). Results remained qualitatively the same (effect of longitudinal age ps<.002, cross-sectional age ps<.004). However, the interaction of longitudinal age and cross-sectional age on round-by round investments was only marginally significant after adjusting for socio-economic status (possibly due to reduced power to detect an interaction effect due to the reduced sample for which socioeconomic data were available), F(1,328)=3.52, p=.06).

Adjusting for IQ, Sex and socio-economic status in the analysis of the effect of family experiences revealed that the interaction of family history and longitudinal age remained significant (interestingly showing a higher effect size than in the unadjusted analysis F(1,6599)=12.21, p<.001)

Brain-behaviour co-development

Association of risk aversion development with anterior insula development

To examine whether there were structural brain correlates for the observed developmental effects on trusting behaviour, we tested for the co-development of cognitive and brain indices in a relatively large

subsample of subjects who underwent two experimental task sessions (baseline and ~18 months) and two structural MRI scan sessions (n=294, see Methods). Recall that in our study social risk aversion emerged as a key cognitive mechanism underlying the development of trust, leading us to hypothesise an association with anterior insula development. This brain region is consistently implicated in trust behaviour, and has been shown to play a role in social risk processing in the trust task [11,12]. Consequently, we extracted grey matter volume values derived from a Voxel Based Morphometry analysis based on peak coordinates suggested by a recent meta-analysis (right Insula: MNI 42, 18, 4) using a 10mm radius [11]. Insula grey matter volume then served as the dependent variable in a mixed model where we adopted the same longitudinal modelling approach detailed for our behavioural analyses. This enabled us to ask whether there were longitudinal and cross-sectional associations between risk aversion and insula grey matter volume, decomposing social risk aversion into its longitudinal and cross-sectional components and entering both as predictors ([14]; See Supplementary Methods). Here, we observed a significant negative effect of age on right insular grey matter volume (F(1,285.26)=20.22, p<.001). Even more striking was evidence for correlated change of insula volume and social risk aversion (F(1,180.51)=4.50, p=.035, S-Figure 8). In effect, a within-person developmental decrease in social risk aversion was also associated with a developmental decrease of insular grey matter volume, over and above the effect of age. See S-Table 8 for full output of the model, including covariates.

Contrary to the specific effects of social risk aversion, correlated change of mean investment behaviour per se and anterior insula development were not statistically significant (F=1, 181.86)=2.60, p=.11). Moreover, a control analysis in a reference region, the ventral striatum, which had been implicated in social reward processing in the trust game, and had been linked to early adversity in the rodent and human literature [17,18], did not reveal any significant associations with trust behaviour, nor family adversity (all ps Fs<1.75, all ps>.187).

Irritability is associated with less age-related change in insula grey matter volume

Given the important role of the Irritability parameter in relation to family adversity, and paralleling our analysis on social risk aversion, we next tested for an association of Irritability with neurodevelopment of right AI whilst accounting for relevant covariates. We found a significant interaction of cross-sectional age with Irritability (F(1,265.64)=11.42, p<.001) on grey matter volume in right AI. As S-Figure 8 B shows, a general age-related decrease in right AI grey matter volume, observed at the group level, was attenuated in those that showed high levels of Irritability across both measurement time points. When

adding family adversity as a moderator, we did not observe any significant main effect of family adversity, nor any significant interaction of family adversity with Irritability on right AI development (all $ps>.41$). Thus, we did not find evidence for an association of family adversity with right AI grey matter development in our generally healthy adolescent population. See S-Table 9 for full output of the model, including covariates.

Computational Modelling: Posterior Predictive Checks

Comparison of simulated vs. empirically observed investment behaviour

We used the computational model along with the individual inferred parameter set to generate a synthetic investment trajectory per individual / measurement time point.
Validity of the model was confirmed by a posterior predictive check analysis, wherein we applied general mixed effect models that were used to predict age/developmental, as well as family adversity effects on investment behaviour, in the empirical data (see Main Methods) to the synthetic (simulated) investment data: This reproduced significant effects of cross-sectional age, longitudinal development as well as the interaction of both factors (all $ps<.002$, see S-Figure 6A). We observed an interaction of childhood family experiences with longitudinal age on investment behaviour, which was, other than in the empirical data, significantly moderated by mean age in the simulated dataset ($F(1,9696.00)=6.17$, $p=.013$, see S-Figure 6B) in a plausible manner; i.e., the strongest difference in trust development as a function of childhood family experiences was present in the youngest of the sample.

In order to map our computational modelling parameter “Irritability”, and inter-individual differences therein, to choice behaviour, we determined a behavioural signature of retaliation: the degree of reduction in own investment behaviour after the participant has observed an unfair trustee action. Unfairness in trustees was defined as a repayment which would leave the investor with less money than the trustee himself after an interaction [19]. According to this definition, n=164 participants in the baseline assessment and n=97 participants in the follow-up assessment did not experience any unfair trustee action and where thus excluded from this proof-of-concept analysis. For the remaining participants, we determined the degree of reduction in investment in trials after an unfair trustee action. As expected, reductions of investments after unfair trustee actions correlated with the computational Irritability parameter (Spearman’s $rho=-.37$, $p<.001$). These investment reductions further served as a dependent variable in a mixed effects model with family adversity, cross-sectional and longitudinal development (and all 2 ways interactions) as predictors. We asked whether such punishment effects predict

investment behaviour (i.e., the raw choice data). A linear mixed effects model predicting investment reductions after unfair actions by cross-sectional age, longitudinal age and family adversity as well as all 2-way interactions revealed no significant developmental or age effects, but showed a significant effect of family adversity on the degree of retaliation, ($b=-0.06$, $SE=0.03$, $F(1,540.15)=3.98$, $p=.047$, $r=.09$, see S-Figure 7), in line with the computational modelling findings.

Supplementary Discussion

Intriguingly, at a neurodevelopmental level, we observed that – over and above the effects of age – a longitudinal decrease in social risk aversion was linked to a decrease in right anterior insula grey matter volume, a region meta-analytic studies have associated with trust, as well as with risk and uncertainty processing in non-social contexts [11,13,20,21]. Anterior insula development during adolescence has also been implicated in the development of cognitive control and a developmental shift in (non-social) risk taking preferences [22]. These neural finding provide a degree of external, construct validity to our behavioural findings and extend the potential developmental significance of anterior insula grey matter change to include the domain of *social* risk preferences. In our sample, irritability estimates were not associated with grey matter insula volume per se, but did act to moderate an age-related decrease of insula grey matter volume. Thus, those with lower irritability estimates showed a normative age-related increase in grey matter volume, whilst this association was not apparent in those with higher irritability estimates. Although not within the primary scope of our investigation, we found no significant effect of family experience on right anterior insula grey matter. One reason might be that here we focused exclusively on right anterior insula as an a priori meta-analytically defined region of interest, based on its relevance for trust and risk behaviour [11-13] whereas longitudinal effects of family environment and parenting have been found predominantly in other regions of the brain, such as the hippocampus [23,24], the amygdala and the OFC [25].

Supplementary Figures

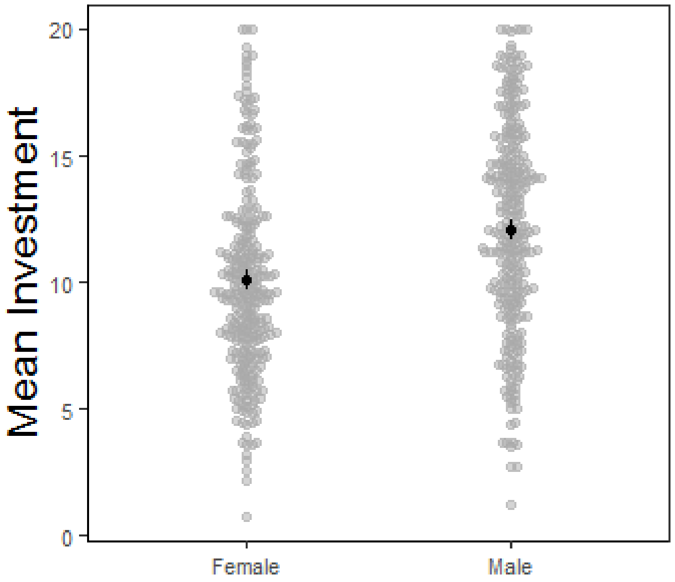

S-Figure 1 Sex Differences in Investment Behaviour (n=570)
Self-reported males invested significantly more than self-reported females, mimicking a pattern we had observed in the baseline (T1) dataset. See [16] for more details. Error bars represent model-based standard errors (plotted using the function afex_plot, R package afex[26])

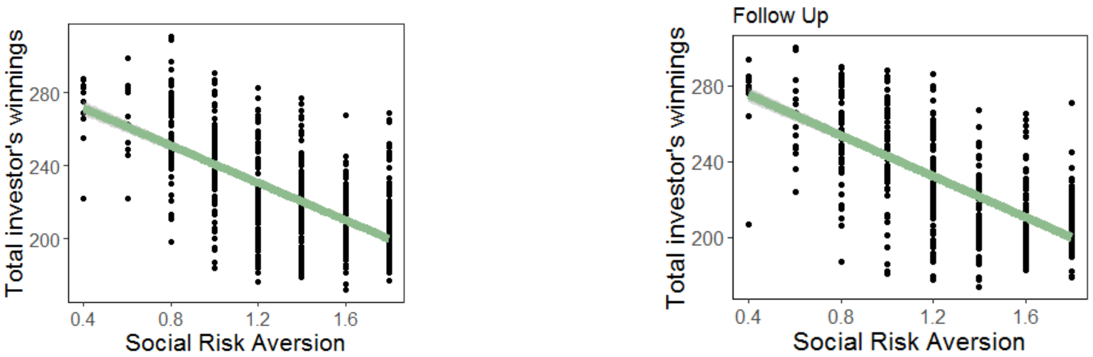

S-Figure 2. Association of social risk aversion with winnings
Social risk aversion is associated with reduced total wins at the end of each testing session, both at T1 and at follow-up.

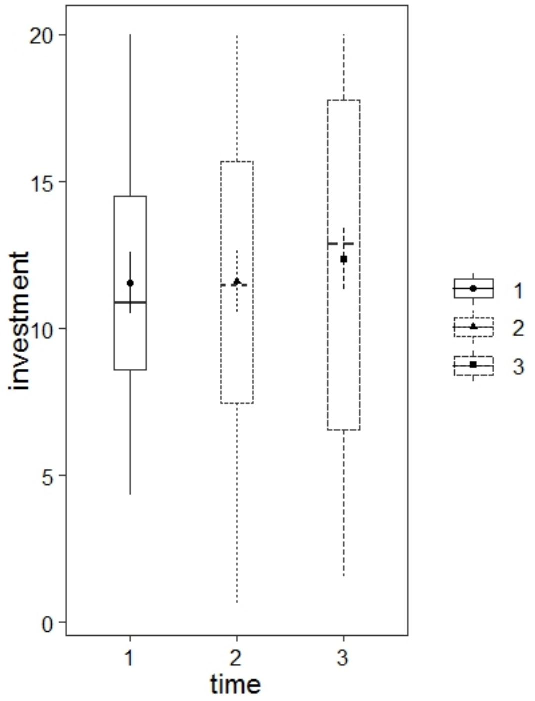

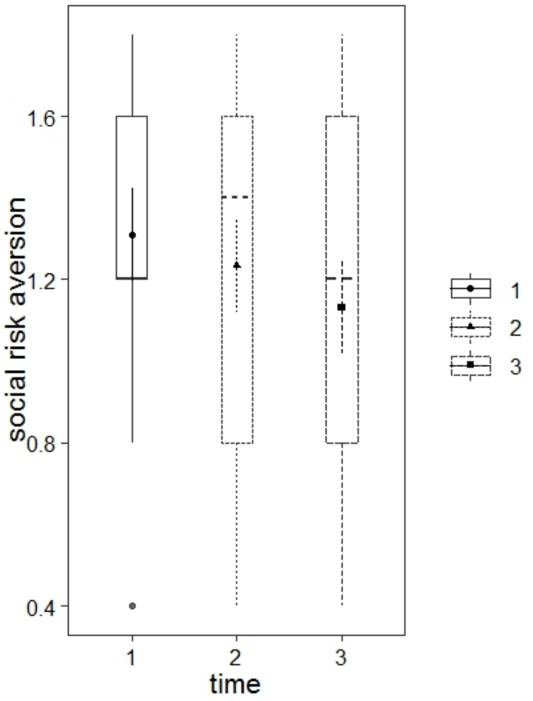

S-Figure 3. In a subsample which underwent 3 measurement time points with our experimental task (n=55), we observed that within-subject change on a) investments and b) the parameter derived from our computational model, social risk aversion, was most pronounced from baseline to the long (1.5 years) follow-up measurement, but was non-significant for the short 6-month follow-up period. This pattern of results speaks in favour of true developmental, rather than training effects. The box in the boxplot represents the middle 50% of the data, the line drawn through the box represents the median, the symbols represent the mean. The lower and upper edges of the box represent the first and third quartiles, respectively

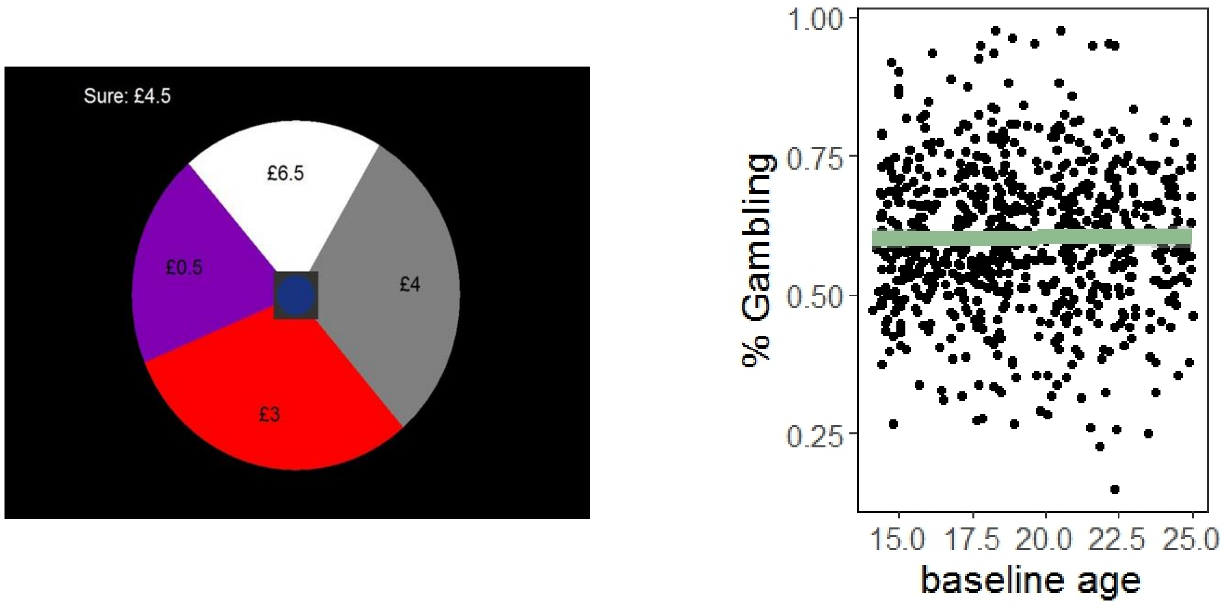

S-Figure 4. No significant association of age at baseline with a tendency to gamble in a traditional, roulette-type risk taking paradigm (see left-hand side for illustration of one trial)[27].

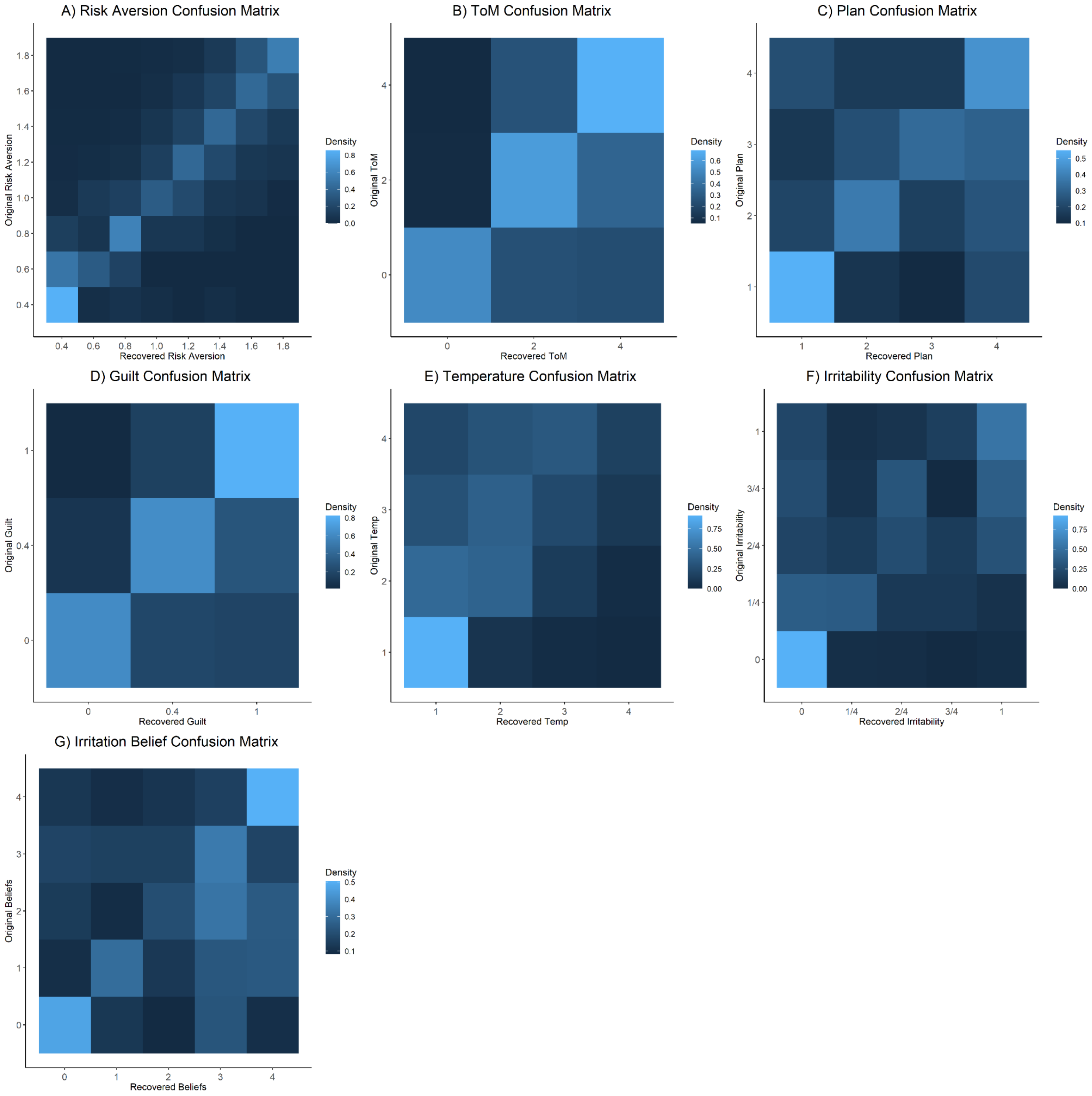

S-Figure 5: Confusion matrix for the recovery analysis of each parameter of the winning computational model. ToM=Theory of Mind.

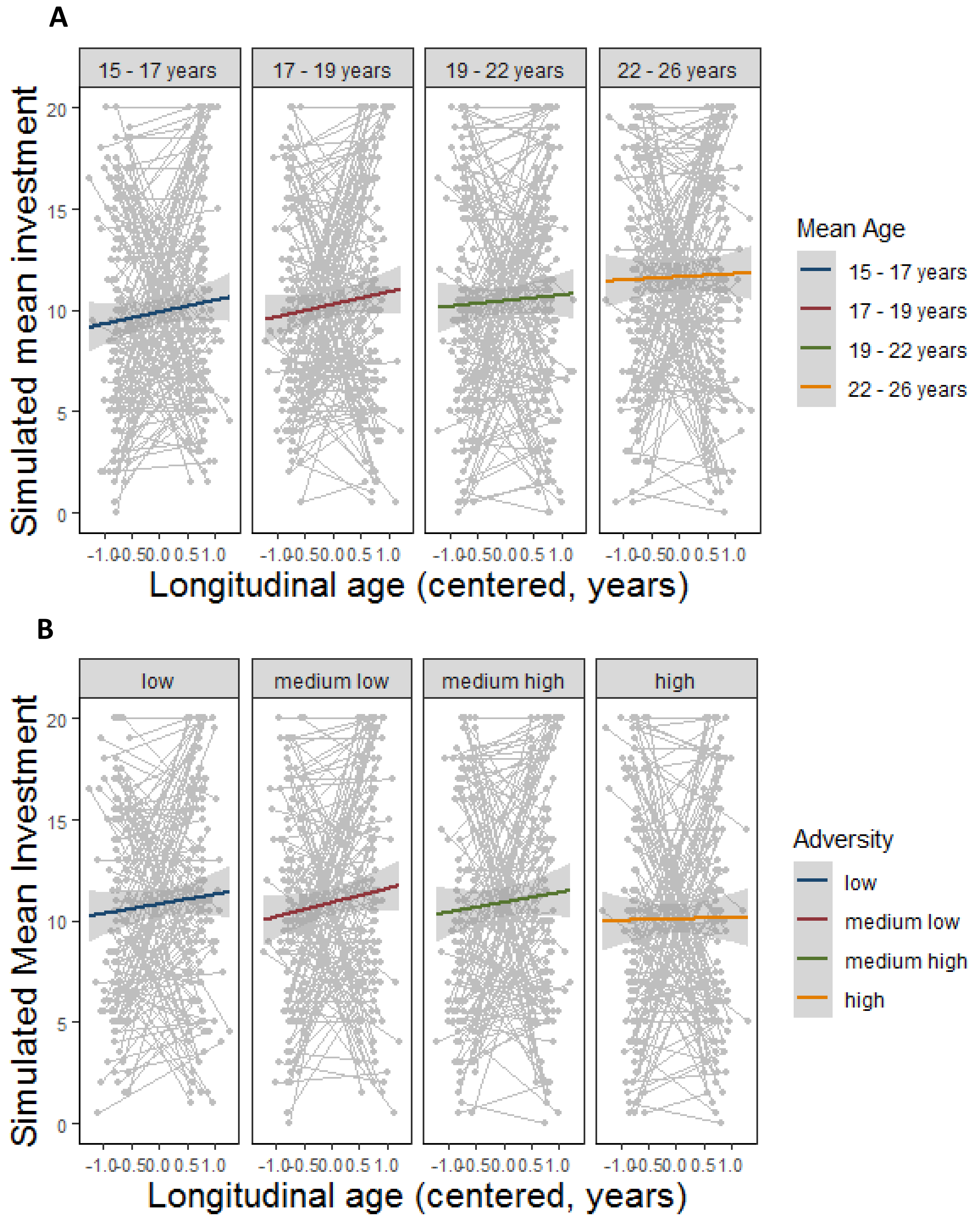

S-Figure 6: Simulated Investment, based on our Computational Trust model. A) The effects of cross-sectional age, longitudinal age and their interaction mimic effects observed on the empirical data. B) Effects of Family Experiences are reproduced on the simulated investment behaviour. Error bands represent the standard error of the mean.

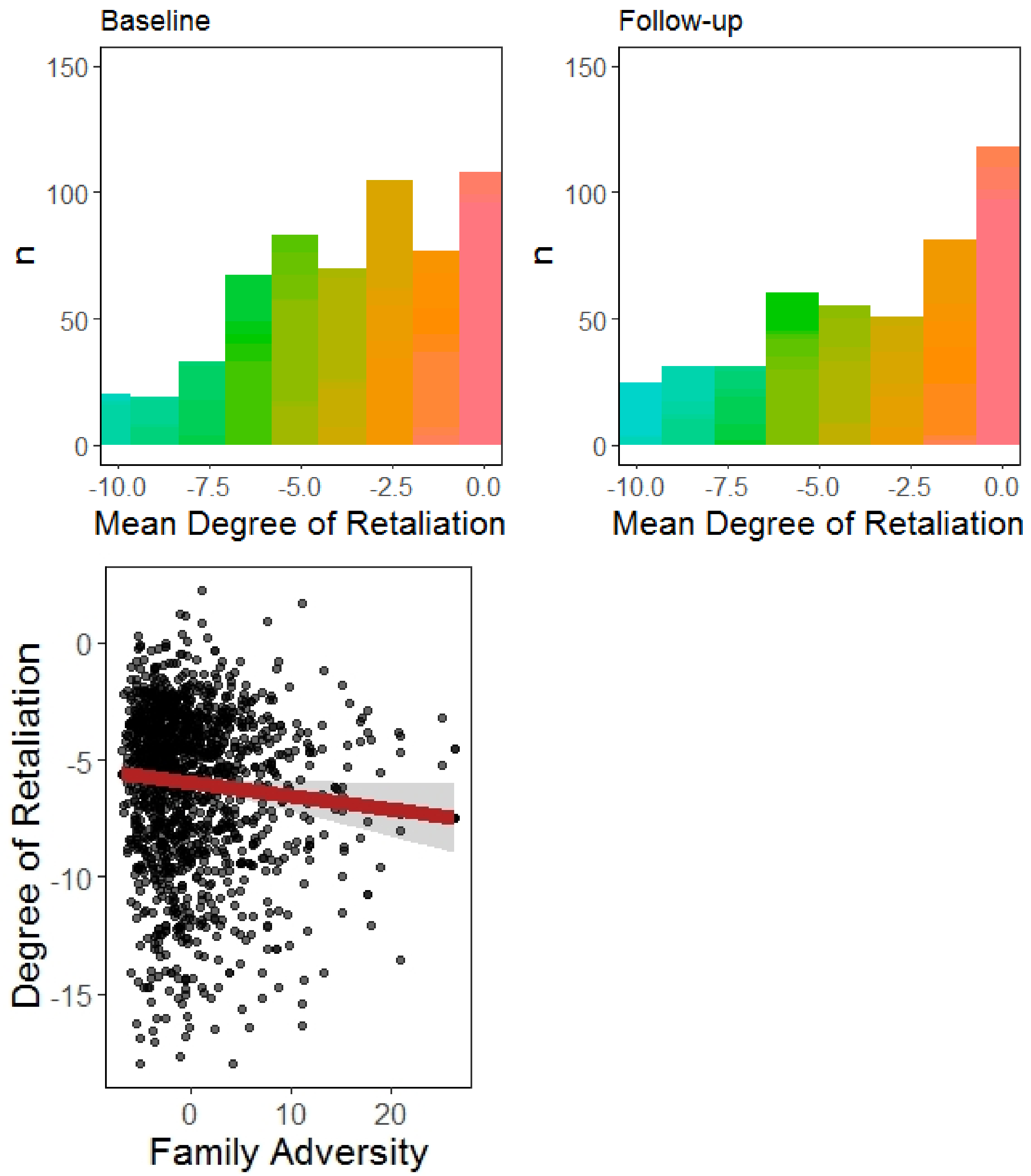

S-Figure 7: Degree of Retaliation as a behavioural measure of our computational irritability parameter at A) Baseline and B) Follow-up. Retaliation was defined as the degree of reduction in investment in trials after an unfair trustee action. Lower values denote a stronger reduction of payment as a response to punishment. C)This reduction was negatively correlated with Family Adversity, thus, those with more adverse family experiences showed a stronger reduction in their own investment in response to an unfair trustee action. Error band represents standard error of the mean.

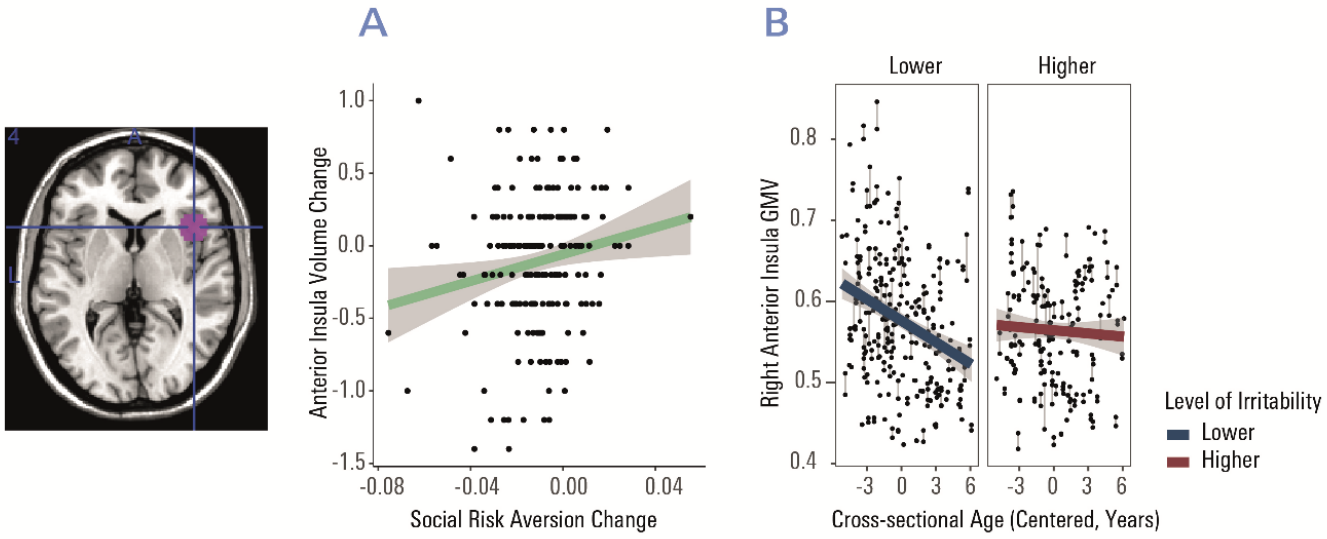

S-Figure 8: Brain Behaviour Co-Development. A) Correlated change of right anterior insula (AI) grey matter volume (GMV) and social risk aversion, indicating that a reduction in social risk aversion is linked to a reduction of insular grey matter volume over the longitudinal follow-up period. B) Significant interaction of age with Irritability on GMV in the right AI ROI. The age-related decrease in AI GMV as observed on the group level was flattened in those with higher Irritability scores. Cross-sectional age is mean centred. Note that whilst Irritability entered the model as a continuous regressor, we categorize it for visualization purposes. Error bands represent standard error of the mean.

Supplementary Tables

S-Table 1

Linear Mixed Effects Model: Effect of longitudinal and cross-sectional age on round 1 investments, adjusted for sex. Test is two-sided. Confidence Intervals are obtained with the function 'confint' (R package lme4)

**Effect**	**df**	**F**	**p**	**CI 2.5%**	**CI 97.5%**
Longitudinal age	1, 568.00	19.45	<.001	.376	.977
Cross-sectional age	1, 567.00	19.10	<.001	.143	.374
Sex	1, 567.00	28.89	<.001	-1.28	-0.59
Longitudinal age x Cross-sectional age	1, 568.00	.32	.572	-0.13	.071

S-Table 2

Linear Mixed Effects Model: Effect of longitudinal and cross-sectional age on trial-by trial investments, adjusted for sex and trial. Test is two-sided. Output of function'nice' (R package afex); Confidence Intervals are obtained with the function 'confint' (R package lme4)

**Effect**	**df**	**F**	**p**	**CI 2.5%**	**CI 97.5 %**
Longitudinal age	1, 10819.00	109.81	<.001	.469	.684
Cross-sectional age	1, 567.00	24.32	<.001	.167	.374
Sex	1, 567.00	49.25	<.001	-1.44	-.816
Trial	9, 10819.00	31.67	<.001	-1.00	-.515
Longitudinal age x Cross-sectional age	1, 10819.00	15.14	<.001	-.11	.0356

S-Table 3

Linear Mixed Effects Model: Effect of longitudinal and cross-sectional age and moderation by family experiences on trial-by trial investments, adjusted for Sex and trial. Test is two-sided. Output of function'nice' (R package afex). Confidence Intervals are obtained with the function 'confint' (R package lme4)

**Effect**	**df**	**F**	**p**	**CI 2.5%**	**CI 97.5 %**
Longitudinal age	1, 9696.00	98.79	<.001	.470	.700
Cross-sectional age	1, 506.00	24.14	<.001	.172	.400
Family History	1, 506.00	2.04	.153	.115	.018
Trial	9, 9696.00	30.63	<.001	-1.078	-.564
Sex	1, 506.00	44.88	<.001	-.147	.806
Longitudinal age x Cross-sectional age	1, 9696.00	5.42	.020	-.085	-.007
Longitudinal age x Family History	1, 9696.00	6.83	.009	-.053	-.007
Cross-sectional age x Family History	1, 506.00	1.57	.211	-.036	.008
Longitudinal age x Cross-sectional age x Family History	1, 9696.00	0.77	.380	.004	.011

S-Table 4

Linear Mixed Effects Model to predict social risk aversion by longitudinal and cross-sectional age , adjusted for sex. Test is two-sided. Output of function 'nice' (R package afex); Confidence Intervals are obtained with the function 'confint' (R package lme4)

**Effect**	**Df**	**F**	**p**	**CI 2.5%**	**CI 97.5 %**
Longitudinal age	1, 568	21.41	<.001	-.079	-.031
Cross-sectional age	1, 567	20.13	<.001	-.030	.012
Sex	1, 567	49.19	<.001	0.068	.121
Longitudinal age x Cross-sectional age	1, 568	2.83 +	.093	.002	.014

S-Table 5

Cumulative Link Mixed Model to predict social risk aversion in an ordinal fashion, fitted with the Laplace approximation (R function 'clmm'). Test is two-sided.

**Effect**	**Estimate**	**z**	**p**
Longitudinal age	-.21477	-4.658	<.001
Cross-sectional age	-.06838	-4.142	<.001
Longitudinal age x Cross-sectional age	.03071	.0152	.04

Threshold coefficients

	Estimate	Std. Error	z value
0.4|0.6	-1.55023	0.09141	-16.959
0.6|0.8	-1.40422	0.08476	-16.568
0.8|1	-0.87440	0.06655	-13.139
1|1.2	-0.35931	0.05759	-6.239
1.2|1.4	0.17319	0.05727	3.024
1.4|1.6	0.77336	0.06465	11.962
1.6|1.8	1.76518	0.08583	20.565

S-Table 6

Linear Mixed Effects Model to predict Irritability. Test is two-sided.

**Effect**	**df**	**F**	**p**	**CI 2.5%**	**CI 97.5 %**
Longitudinal age	1, 507.00	.49	.482	-.016	-.035
Cross-sectional age	1, 506.00	.56	.453	-.009	.004
Family History	1, 506.00	7.92	.005	.002	.010
Sex	1, 506.00	1.27	.260	-.033	.009
Longitudinal age x Cross-sectional age	1, 507.00	1.27	.261	-.004	0.014
Longitudinal age x Family History	1, 507.00	1.19	.276	-.002	.007
Cross-sectional age x Family History	1, 506.00	.30	.585	-.002	.001
Longitudinal age x Cross-sectional age x Family History	1, 507.00	.01	.93	-.002	.002

S-Table 7

Cumulative Link Mixed Model to predict irritability in an ordinal fashion, fitted with the Laplace approximation (R function clmm, link). Test is two-sided.

Effect	Estimate	Z	p
Longitudinal age	-0.21477	-4.658	.734
Family History	.0377587	2.747	.006
Cross-sectional age	-.06838	-1.467	.1423
Sex	.0526	.355	.722
Longitudinal age x Cross-sectional age	.0215	.726	.467
Cross-sectional age x Family History	-.0008	-.172	.863
Longitudinal age x Cross-sectional age x Family History	-.002	-.355	0.722

Threshold coefficients:

	Estimate	Std. Error	z value
0|0.25	1.4928	0.1252	11.92
0.25|0.5	1.9140	0.1370	13.97
0.5|0.75	2.3132	0.1497	15.45
0.75|1	2.5533	0.1581	16.15

S-Table 8

Effect of risk aversion, longitudinal and cross-sectional age on Grey Matter Volume in the Right Anterior Insula R OI. Test is two-sided.

Effect	df	F	p	CI 2.5%	CI 97.5 %
Risk Aversion longitudinal	1, 180.51	4.50	.035	.0004	.011
Age longitudinal	1, 231.03	.00	.965	-.008	.008
Risk Aversion cross-sectional	1, 281.04	.54	.463	.014	.031
Age cross-sectional	1, 283.97	21.36	<.001	-.009	.004
Movement Regressor	1, 205.87	.05	.827	-.006	.005
Sex	1, 359.87	15.76	<.001	-.027	-.009
Ethnicity	1, 234.40	3.42 +	.066	-.0002	.010
Total intracranial Volume	1, 460.59	44.99	<.001	.0001	.0002
Scanning Site1	1, 443.23	.56	.453	-.012	.005
Scanning Site 2	1, 201.05	6.48	.012	.001	.008
Risk Aversion cross-sectional x Age longitudinal	1, 180.16	.12	.725	-.004	.006
Risk Aversion cross-sectional x Age cross-sectional	1, 281.39	.15	.695	-.006	.009

S-Table 9

Linear Mixed Effects Model:
Effect of Irritability, longitudinal and cross-sectional age on Grey Matter Volume in the Right Anterior Insula ROI. Test is two-sided.

**Effect**	**df**	**F**	**p**	**CI 2.5%**	**CI 97.5 %**
Irritability longitudinal	1, 180.45	1.60	.208	-.002	.009
Irritability cross-sectional	1, 282.35	.03	.874	-.025	.029
Age longitudinal	1, 233.51	.03	.865	-.008	.007
Age cross-sectional	1, 284.14	24.58	<.001	-.009	-.003
Movement Regressor	1, 207.56	.19	.664	-.006	.004
Sex	1, 363.89	15.49	<.001	-.026	-.009
Ethnicity	1, 236.91	3.33	.069	-.0003	.010
Total Intracranial Volume	1, 464.21	49.84	<.001	.0001	.0002
Scanning Site1	1, 438.05	.45	.502	-.012	.005
Scanning Site 2	1, 202.55	4.96	.027	.0005	.008
Irritability crosssectional x Age longitudinal	1, 180.76	.26	.612	-.005	.008
Irritability crosssectional Age crosssectional	1, 282.83	9.31	.003	-.006	.010

S-Table 10

*Linear Mixed Effects Model***:** *Effect of Round 1 Investment (A priori Trust), family experiences, longitudinal age and cross-sectional age on friendships (from T1q until T3q), adjusted for Sex. Test is two-sided.*

**Effect**	**df**	**F**	**p**	**CI 2.5%**	**CI 97.5 %**
Trial 1 investment	1, 677.03	4.31	.038	.003	.010
Longitudinal age	1, 1050.70	40.33	<.001	.315	.60
Family Experiences	1, 698.25	73.88	<.001	-.250	-.158
Cross-sectional age	1, 681.36	5.98	.015	.020	.174
Sex	1, 683.07	4.03	.045	.007	.461
Trial 1 investment x longitudinal age	1, 1050.70	6.34	.012	.008	.064
Trial 1 investment x Family Experiences	1, 698.57	0.03	.854	-.009	.011
Longitudinal age x Family Experiences	1, 1050.70	13.63	<.001	.024	.077
Trial 1 investment x Family Experiences	1, 682.63	0.82	.364	-.022	.008
Longitudinal age x Cross-sectional age	1, 1050.70	16.15	<.001	-.015	-.050
Family Experiences x Cross-sectional age	1, 704.14	6.88	.009	.005	.036
Trial 1 investment x Longitudinal age x Family Experiences	1, 1050.70	6.77	.009	-.002	.014
Trial 1 investment x Longitudinal age x Cross-sectional age	1, 1050.70	0.35	.554	-.006	.012
Trial 1 investment x Family Experiences x Cross-sectional age	1, 724.17	1.91	.168	.001	.006

S-Table 11

Neuroscience in Psychiatry Network (NSPN) Consortium author list

**NSPN Principle Investigators**
Edward Bullmore<br>Raymond J. Dolan<br>Ian M. Goodyer<br>Peter Fonagy<br>Peter Jones
**NSPN (funded) staff:**
Michael Moutoussis<br>Tobias U. Hauser<br>Sharon Neufeld<br>Rafael Romero-Garcia<br>Michelle St Clair<br>Petra Vértes<br>Kirstie Whitaker<br>Becky Inkster<br>Gita Prabhu<br>Cinly Ooi<br>Umar Toseeb<br>Barry Widmer<br>Junaid Bhatti<br>Laura Villis<br>Ayesha Alrumaithi<br>Sarah Birt<br>Aislinn Bowler<br>Kalia Cleridou<br>Hina Dadabhoy<br>Emma Davies<br>Ashlyn Firkins<br>Sian Granville<br>Elizabeth Harding<br>Alexandra Hopkins<br>Daniel Isaacs<br>Janchai King<br>Danae Kokorikou<br>Christina Maurice<br>Cleo McIntosh<br>Jessica Memarzia<br>Harriet Mills<br>Ciara O’Donnell<br>Sara Pantaleone<br>Jenny Scott<br>Matilde Vaghi<br>Andrea M.F. Reiter<br>Lucy Vanes

Supplementary References

1 King-Casas, B. *et al.* Getting to know you: reputation and trust in a two-person economic exchange. *Science* **308**, 78-83 (2005).

2 Fett, A.-K. J. *et al.* To trust or not to trust: the dynamics of social interaction in psychosis. *Brain* **135**, 976-984 (2012).

3 Essau, C. A., Sasagawa, S. & Frick, P. J. Psychometric properties of the Alabama parenting questionnaire. *Journal of Child and Family Studies* **15**, 595-614 (2006).

4 Elgar, F. J., Waschbusch, D. A., Dadds, M. R. & Sigvaldason, N. Development and validation of a short form of the Alabama Parenting Questionnaire. *Journal of Child and Family Studies* **16**, 243-259 (2007).

5 van Harmelen, A. L. *et al.* Adolescent friendships predict later resilient functioning across psychosocial domains in a healthy community cohort. *Psychol Med* **47**, 2312-2322, doi:10.1017/S0033291717000836 (2017).

6 Kiddle, B. *et al.* Cohort profile: the NSPN 2400 Cohort: a developmental sample supporting the Wellcome Trust NeuroScience in Psychiatry Network. *International journal of epidemiology* **47**, 18-19g (2017).

7 Weiskopf, N. *et al.* Quantitative multi-parameter mapping of R1, PD*, MT, and R2* at 3T: a multi-center validation. *Frontiers in neuroscience* **7**, 95 (2013).

8 Helms, S. W. *et al.* Adolescents misperceive and are influenced by high-status peers' health risk, deviant, and adaptive behavior. *Developmental psychology* **50**, 2697 (2014).

9 Ashburner, J. A fast diffeomorphic image registration algorithm. *Neuroimage* **38**, 95-113 (2007).

10 Draganski, B. *et al.* Regional specificity of MRI contrast parameter changes in normal ageing revealed by voxel-based quantification (VBQ). *Neuroimage* **55**, 1423-1434 (2011).

11 Bellucci, G., Chernyak, S. V., Goodyear, K., Eickhoff, S. B. & Krueger, F. Neural signatures of trust in reciprocity: A coordinate-based meta-analysis. *Human brain mapping* **38**, 1233-1248 (2017).

12 Bellucci, G., Feng, C., Camilleri, J., Eickhoff, S. B. & Krueger, F. The role of the anterior insula in social norm compliance and enforcement: Evidence from coordinate-based and functional connectivity meta-analyses. *Neuroscience & Biobehavioral Reviews* **92**, 378-389 (2018).

13 Mohr, P. N., Biele, G. & Heekeren, H. R. Neural processing of risk. *Journal of Neuroscience* **30**, 6613-6619 (2010).

14 Vanes, L. D. *et al.* White matter tract myelin maturation and its association with general psychopathology in adolescence and early adulthood. *Hum Brain Mapp* **41**, 827-839, doi:10.1002/hbm.24842 (2020).

15 Ziegler, G. *et al.* Compulsivity and impulsivity traits linked to attenuated developmental frontostriatal myelination trajectories. *Nat Neurosci* **22**, 992-999, doi:10.1038/s41593-019-0394-3 (2019).

16 Hula, A. *et al.* Multi-Round Trust Game quantifies inter-individual differences in Social Exchange from Adolescence to Adulthood. *Computational Psychiatry* (2021).

17 Hanson, J. L., Williams, A. V., Bangasser, D. A. & Peña, C. J. Impact of early life stress on reward circuit function and regulation. *Frontiers in Psychiatry*, 1799 (2021).

18 Fareri, D. S. & Tottenham, N. Effects of early life stress on amygdala and striatal development. *Developmental cognitive neuroscience* **19**, 233-247 (2016).

19 Fehr, E. & Schmidt, K. M. A theory of fairness, competition, and cooperation. *The quarterly journal of economics* **114**, 817-868 (1999).

20 Bach, D. R. & Dolan, R. J. Knowing how much you don't know: a neural organization of uncertainty estimates. *Nature reviews neuroscience* **13**, 572-586 (2012).

21 Preuschoff, K., Quartz, S. R. & Bossaerts, P. Human insula activation reflects risk prediction errors as well as risk. *The Journal of neuroscience : the official journal of the Society for Neuroscience* **28**, 2745-2752, doi:10.1523/JNEUROSCI.4286-07.2008 (2008).

22 Smith, A. R., Steinberg, L. & Chein, J. The role of the anterior insula in adolescent decision making. *Developmental neuroscience* **36**, 196-209 (2014).

23 Luby, J. L. *et al.* Maternal support in early childhood predicts larger hippocampal volumes at school age. *Proceedings of the National Academy of Sciences* **109**, 2854-2859 (2012).

24 Rao, H. *et al.* Early parental care is important for hippocampal maturation: evidence from brain morphology in humans. *Neuroimage* **49**, 1144-1150 (2010).

25 Whittle, S. *et al.* Positive parenting predicts the development of adolescent brain structure: a longitudinal study. *Developmental cognitive neuroscience* **8**, 7-17 (2014).
26 Singmann, H. *et al.* (2016).
27 Bach, D. R., Moutoussis, M., Bowler, A. & Dolan, R. J. Predictors of risky foraging behaviour in healthy young people. *Nature human behaviour* **4**, 832-843 (2020).
