# Supplementary material for: Self-reported childhood family adversity is linked to an attenuated gain of trust during adolescence
Source: Nat Commun. 2023 Oct 30;14:6920. doi: 10.1038/s41467-023-41531-z (PMC10616102; doi:10.1038/s41467-023-41531-z)
Supplement: Supplementary file 2 — Reporting Summary [file 41467_2023_41531_MOESM2_ESM.pdf]

nature portfolio

Corresponding author(s): Andrea Reiter
Last updated by author(s): May 15, 2023

Reporting Summary

Nature Portfolio wishes to improve the reproducibility of the work that we publish. This form provides structure for consistency and transparency in reporting. For further information on Nature Portfolio policies, see our Editorial Policies and the Editorial Policy Checklist.

Statistics

For all statistical analyses, confirm that the following items are present in the figure legend, table legend, main text, or Methods section.

n/a	Confirmed	
☐	☒	The exact sample size (*n*) for each experimental group/condition, given as a discrete number and unit of measurement
☐	☒	A statement on whether measurements were taken from distinct samples or whether the same sample was measured repeatedly
☐	☒	The statistical test(s) used AND whether they are one- or two-sided<br>*Only common tests should be described solely by name; describe more complex techniques in the Methods section.*
☐	☒	A description of all covariates tested
☐	☒	A description of any assumptions or corrections, such as tests of normality and adjustment for multiple comparisons
☐	☒	A full description of the statistical parameters including central tendency (e.g. means) or other basic estimates (e.g. regression coefficient) AND variation (e.g. standard deviation) or associated estimates of uncertainty (e.g. confidence intervals)
☐	☒	For null hypothesis testing, the test statistic (e.g. *F*, *t*, *r*) with confidence intervals, effect sizes, degrees of freedom and *P* value noted<br>*Give P values as exact values whenever suitable.*
☒	☐	For Bayesian analysis, information on the choice of priors and Markov chain Monte Carlo settings
☒	☐	For hierarchical and complex designs, identification of the appropriate level for tests and full reporting of outcomes
☐	☒	Estimates of effect sizes (e.g. Cohen's *d*, Pearson's *r*), indicating how they were calculated

Our web collection on statistics for biologists contains articles on many of the points above.

Software and code

Policy information about availability of computer code

Data collection	Behavioural data were acquired using MATLAB 2012a and the Cogent graphic toolbox (COGENT 2000), http://www.vislab.ucl.ac.uk/Cogent/)
Data analysis	Statistical analyses were conducted using R (Version 4.0.3) and Rstudio (Version 1.1.383) using the following packages:<br>afex (Version 0.28.1) ordinal (Version 2019.12.10) and emmeans (Version 1.6.0). Plots were generated using ggplot2 (Version 3.3.3).<br>Computational Modeling code was written and model fitting was done in C++ using custom code (https://github.com/AndreasHula/TrustGameCPSY and https://github.com/AndreasHula/BacktestingTrustNSPNBaseline). Semiquantitative MT saturation maps were derived using the hMRI toolbox (www.hmri. info) for SPM (Wellcome Centre for Human Neuroimaging, London, UK, http://www.fil.ion.ucl.ac.uk/spm).<br>Whole-brain maps were prepocessed using the Computational Anatomy Toolbox (http://www.neuro.uni-jena.de/cat/).

For manuscripts utilizing custom algorithms or software that are central to the research but not yet described in published literature, software must be made available to editors and reviewers. We strongly encourage code deposition in a community repository (e.g. GitHub). See the Nature Portfolio guidelines for submitting code & software for further information.

Data

Policy information about availability of data

All manuscripts must include a data availability statement. This statement should provide the following information, where applicable:

- Accession codes, unique identifiers, or web links for publicly available datasets
- A description of any restrictions on data availability
- For clinical datasets or third party data, please ensure that the statement adheres to our policy

All anonymized data are available via the Open Science Framework https://osf.io/42b68/

Research involving human participants, their data, or biological material

Policy information about studies with human participants or human data. See also policy information about sex, gender (identity/presentation), and sexual orientation and race, ethnicity and racism.

Reporting on sex and gender	When signing up for this study, participants were asked to tick: Sex: 'Female' or 'Male'. It was not clarified if some understood the question as 'gender identity', socially attributed or biological category. Due to the phrasing 'Sex:' one might speculate that most participants understood the question to mean 'self-reported estimate of biological sex', which is why we use the term 'sex' in this article, even though we do not have disaggregated information on participants' sex vs. gender.<br>We use self-reported sex (f/m) as a covariate in all analyses, and report effects of self-reported sex in the Supplementary Information File (also see our previous publication, Hula et al., 2021 Computational Psychiatry for a paper that focuses on, and discusses, sex differences in the baseline data of the reported dataset) .
Reporting on race, ethnicity, or other socially relevant groupings	Self-reported ethnicity was assessed in NSPN but not considered as a covariate in the behavioural analyses, as we did not have hypotheses about any differences in trusting behaviour as a function of ethnicity.<br>As is common in structural MRI analysis, self-reportd ethnicity was included into the MRI model as a covariate.
Population characteristics	Data for this task was available from n=570 (285 female) participants for baseline and follow-up. Participants were 14.10-24.99 years old (mean=19.05, sd=2.96) at baseline. Mean age at follow-up was 20.30 years (range: 15.11-26.48 years, sd=2.98). Mean time between first and second task assessment was 1.48 years (range: 0.99-2.6 years, sd = 0.29). Structural imaging and task data were available (and passed quality assessment) for n=294 participants. A subgroup of participants (n=55) underwent the task three times, allowing for an analysis of retest effects (see SI Methods). Participants were part of the larger NSPN Cohort which recruited >2000 participants in an age- sex-stratified sample, including equal numbers of males and females for the following five age groups: 14-15, 16-17, 18-19, 20-21, and 22-24.99years. See Kiddle et al., 2018, Int J Epidemiol , for more information.
Recruitment	The NIHR Primary Care Research Network (PCRN) engaged 50 GPs to recruit young people using their sex-age registers by sending out invitations (including an expressions of interest form (EoI)) across Cambridgeshire and Greater London (closest proximity to universities leading the study). Schools and Further Education colleges were also engaged to distribute the EoI forms to 14 to 18-year-old participants. The NSPN recruitment team assisted GPs and schools by providing invitation to participate letters, which were forwarded to potential participant's home address that remained unknown to the NSPN investigators. Purposive advertisement was also used during recruitment; invitation letters with EoI were sent to those who responded to advertisements that met the age criteria. If an individual wanted to participate they informed NSPN recruitment team over the phone/sent in completed EoI form. See also Kiddle et al., 2018, Int J Epidemiol , for more information (including a STROBE diagram: Fig 2).<br>A key self-selection bias that we identified was a differential self-selection into the 'cognitive cohort' and the 'scanning cohort' on the basis of socio-economic status and an interaction of self-reported gender and general cognitive ability, as measured by IQ. Namely, we observed that more male participants of higher IQ self-selected for the cognitive cohort; and that fewer young people of low socioeconomic status, compared to England as a whole, selected themselves for both the cognitive and MRI cohorts. We also repeat that the study samples focuses on the healthy population by construction. A more detailed analysis of demographic differences between the baseline population, based on census data, and the analysed samples can be found in Figure S2 of the Supplement of Ziegler at al, 2020 Human Brain Mapping. We note that such self selection biases (e.g. in terms of socio economic class or cognitive abilities) are likely to be very common in published studies in Developmental Psychology/Neuroscience. We do not believe that these biases are likely to affect our analyses, for example, we took care to examine the effects of cross-sectional age, self-declared sex and IQ, and socioeconomic class . However, we cannot exclude that these self-selection and related biases limit the generalizability of our results.
Ethics oversight	All participants provided written informed consent. The Cambridge Central Research Ethics Committee approved the study (12/EE/0250).

Note that full information on the approval of the study protocol must also be provided in the manuscript.

Field-specific reporting

Please select the one below that is the best fit for your research. If you are not sure, read the appropriate sections before making your selection.

☐ Life sciences ☒ Behavioural & social sciences ☐ Ecological, evolutionary & environmental sciences

For a reference copy of the document with all sections, see nature.com/documents/nr-reporting-summary-flat.pdf

Behavioural & social sciences study design

All studies must disclose on these points even when the disclosure is negative.

Study description	This is a longitudinal, quantitative study employing an experimental task, computational modeling, structural neuro-imaging as well as self-report questionnaires to probe the development of trust in adolescence , its neural and computational correlates and the influence of family adversity.
Research sample	The sample consists of 570 participants (14-25 years of age, 284 female, 286 male). These are part of a larger cohort study: https://nspn.org.uk/ ('NSPN u change cognition cohort'). See Kiddle et al., 2018, Int J Epidemiol for a complete description of the cohort.<br>A 'cognitive cohort' was formed by sampling participants from a pool of around 2400 community-dwelling young people. Participants were randomly contacted from 5 different age groups (14-16, 16-18, etc.) until each age group had roughly equal proportions of both males and females. The proportion of non-white-English participants in the study was within 10% of the most recent census data. To ensure that the sample was representative of the healthy population, significant neuropsychiatric issues were screened out by self-report, and recruitment sources were selected accordingly. The cognitive cohort was expanded until it reached 780 participants (for baseline assessment), of whom 300 were invited for MRI brain scanning, with an equal number of males and females across the five age groups.
Sampling strategy	Sample size was chosen to extend previous studies which reported age/developmental effects on trust in adolescence: van den Bos et al (2010) Cognitive Development; Westhoff B et al (2020) Scientific reports; van de Groep et al (2020) Journal of Research on Adolescence ; Lemmers-Jansen et al (2017) Developmental cognitive neuroscience; Fett A-KJ, et al. (2014) Journal of Adolescence. articipants were randomly contacted from a pool of 2400 young individuals. See Kiddle et al., 2018, Int J Epidemiol for a complete description of the cohort.
Data collection	Data were collected by trained RAs, using computerized tasks in the lab. RAs were blind to the specific hypotheses underlying this study. Only RA and participant were present during data collection.
Timing	Data were collected between 2012 and 2017
Data exclusions	No behavioural data were excluded. MRI scans were excluded after visual and quantitative inspection for quality assessment (e.g. due to excessive head motion).
Non-participation	For the task analysed, n=570 from originally n= 784 participants completed the follow up. This corresponds to a retunr rate of ~73%, which is comparable to attrition rates of other European large-scale longitudinal studies in adolescence (e.g. the IMAGEN consortium). We have only anectdotal insight into why participants chose not to return for the follow-up. In this developmental period, reasons likely include geographical relocation or embarking on new educational courses/employment.
Randomization	Participants were not allocated to experimental groups

Reporting for specific materials, systems and methods

We require information from authors about some types of materials, experimental systems and methods used in many studies. Here, indicate whether each material, system or method listed is relevant to your study. If you are not sure if a list item applies to your research, read the appropriate section before selecting a response.

Materials & experimental systems

n/a	Involved in the study	
☒	☐	Antibodies
☒	☐	Eukaryotic cell lines
☒	☐	Palaeontology and archaeology
☒	☐	Animals and other organisms
☒	☐	Clinical data
☒	☐	Dual use research of concern
☒	☐	Plants

Methods

n/a	Involved in the study	
☒	☐	ChIP-seq
☒	☐	Flow cytometry
☐	☒	MRI-based neuroimaging

Magnetic resonance imaging

Experimental design

Design type	structural MRI
Design specifications	N/A (no task-based MRI)
Behavioral performance measures	N/A (no task-based MRI)

Acquisition

Imaging type(s): structural

Field strength: 3

Sequence & imaging parameters: Multi-echo FLASH Magnetization Transfer (MT) with TR: 23.7; flip angle = 6; 176 sagittal slices; FOV=256 x 240 mm2; matrix = 256 x 240 x 176; 1mm isotropic

Area of acquisition: whole-brain

Diffusion MRI: ☐ Used ☒ Not used

Preprocessing

Preprocessing software: We used SPM 12 (Wellcome Centre for Human Neuroimaging, London, UK, http://www.fil.ion.ucl.ac.uk/spm). for pre-processing. Maps were segmented and normalized using the Computational Anatomy Toolbox (http://www.neuro.uni-jena.de/cat/).

Normalization: affine, nonlinear (geodesic shooting)

Normalization template: MNI

Noise and artifact removal: cans were manually inspected by an expert for motion artefacts or segmentation failure. A statistical covariance-based inhomogeneity measure (implemented in the CAT toolbox) was also used to detect and remove subjects with extreme overall deviation of quantitative values. Lastly, a proxy of in-scanner motion (SD parameter of the R2* exponential decay residuals; SDR2*) was calculated for each subject and those with SDR2* values above 2.5 SD from the sample mean were excluded. SDR2* values were also used as covariates in the statistical model to analyze grey matter volume (see below).

Volume censoring: N/A (no functional MRI)

Statistical modeling & inference

Model type and settings: Mean grey matter volume (GMV) was extracted from the peak coordinates found in the meta-analysis (right AI MNI 44, 2, -42; 10mm sphere). These grey matter volume values were then entered into a mixed model as dependent variable, and predicted by social risk aversion (one regressor indexing cross-sectional (between-subject differences) and longitudinal (within-subject change) variance, respectively), as well as cross-sectional and longitudinal age. Additionally, two-way interactions of mean ("cross-sectional") social risk-aversion and both age components were included as predictors (see Ziegler et al, 2019, Nature Neuroscience for a similar analytical approach in the same dataset). The same model was subsequently set up for the Irritability parameter, additionally including family adversity, following up on our behavioural results.
Movement, sex, ethnicity, total intracranial volume and scanning site were included as nuisance regressors .

Effect(s) tested: A mixed effects mode was set-up as follows:
Right AI Grey Matter Volume ROI ~ model parameter longitudinal + model parameter cross-sectional + age longitudinal + age cross-sectional + model parameter cross- sectional x age longitudinal + model parameter cross-sectional x age cross-sectional + movement + sex + ethnicity + tiv + scanning site + (1|Participant)

Specify type of analysis: ☐ Whole brain ☒ ROI-based ☐ Both

Anatomical location(s): A recent coordinate-based meta-analysis (Bellucci et al., 2017, Human Brain Mapping), based on 23 original publications reporting on fMRI during economic trust games, found that decisions to trust most consistently involved anterior insula (AI). Mean grey matter volume (GMV) was thus extracted from the peak coordinates found in the meta-analysis (right AI MNI 44, 2, -42), using a 10mm radius.

Statistic type for inference (See Eklund et al. 2016): Mean GMV values from within this mask region were extracted from each map and entered into a hierarchical mixed effects model

Correction: Mean GMV values from within this mask region were extracted from each map and entered into a hierarchical mixed effects model

Models & analysis

n/a	Involved in the study
☒	☐ Functional and/or effective connectivity
☒	☐ Graph analysis
☒	☐ Multivariate modeling or predictive analysis
